# Gambling in the Shadow of War: Evidence of Increased Problem Gambling for Men with Difficulties in Emotional Regulation During a Mass Trauma

**DOI:** 10.1007/s10899-025-10417-8

**Published:** 2025-09-09

**Authors:** Uri Lifshin, Hagit Bonny-Noach, Vera Skvirsky, Dvora Shmulewitz, Merav Vider, Ariel Kor, Shaul Lev-Ran, Mario Mikulincer

**Affiliations:** 1 Department of Psychology and Azrieli Israel Center for Addiction and Mental Health, The Hebrew University of Jerusalem, Jerusalem, Israel; 2 Department of Criminology, Ariel University, Ariel, Israel; 3 Faculty of Medicine and Azrieli Israel Center for Addiction and Mental Health, The Hebrew University of Jerusalem, Jerusalem, Israel; 4 Lev Hasharon Medical Center, Netanya, Israel; 5 Faculty of Medicine and Health Sciences, Tel Aviv University, Tel Aviv, Israel

**Keywords:** Addiction, Emotion regulation, Gambling, Trauma, War

## Abstract

Research suggests that Problem Gambling (PG) may result from maladaptive emotional regulation strategies aimed at escaping emotions and stress caused by negative life events and trauma. The current study, aimed to examine the effect of exposure to the mass trauma of October 7, 2023 terror attack and the consequent Swords of Iron war in Israel, and difficulties in emotional regulation on PG. We utilized longitudinal data of self-reports in the Problem Gambling Severity Index collected before the terror attack (April 2022) and during the Swords of Iron war (December 2023, March 2024, June 2024; *N* = 899) from a large sample of adult Jewish Israelis. Participants also completed the Difficulties in Emotional Regulation Scale before the war (April 2022). Results indicated that for non-problem gamblers, among men, high difficulties in emotion regulation scores were associated with increased PG scores from the time before to the times during the war (*b*s > 0.53, *p-value*s < 0.003) but not for women or men with low difficulties in emotion regulation scores (*b*s < 0.36, *p-value*s > 0.167). Among participants at moderate risk for PG and problem gamblers, there was a decrease in PG scores, especially among participants low in difficulties in emotion regulation, beyond gender. Findings highlight the important role of collective traumatic events and difficulties in emotion regulations as predictors of problem gambling. We discuss the study limitations, including the relatively small number of participants at risk for PG and the specificity of the sample, and offer directions for future research.

**Supplementary Information:**

The online version contains supplementary material available at 10.1007/s10899-025-10417-8.

## Introduction

Gambling is an ancient human endeavor that can be harmless, enjoyable and even beneficial for many people (e.g., Calado & Griffiths, 2016). However, gambling can also lead to negative consequences for the self and others (e.g., Hodgins & Stinchfield, 2008). Problem gambling (PG) refers to gambling that disrupts or damages personal, family or recreational pursuits (e.g., Goodwin et al., 2017; Lesieur & Rosenthal, 1991). Research consistently indicated that PG can lead to significant personal and social harm, such as physical and mental health problems, as well as financial, relationship, and legal problems (e.g., Hodgins & Stinchfield, 2008; Lesieur & Rosenthal, 1991).

A recent representative study conducted in Israel found that about 16.7% of the sample reported low-risk and above in problem gambling (Gavriel-Fried et al., 2023a). In most countries and cultures, including Israel, men tend to engage in gambling more than women (e.g., Allami et al., 2021; Bonny-Noach, 2024; Husky et al., 2015; Gavriel-Fried et al., 2023b; Stark et al., 2012; Tran et al., 2024). This may be due to societal norms making gambling more acceptable and accessible for men (e.g., Holdsworth et al., 2012; Man & Cheung, 2022; McMillen et al., 2004). Gender differences in PG may also result from differences in the psychological processes underlying PG (e.g., Holdsworth et al., 2012; Merkouris et al., 2016). Research indicates that men and women may employ different strategies for managing emotions as men are more likely to use suppression and avoidance, while women tend to engage in strategies like seeking social support and emotional expression (Nolen-Hoeksema, 2012). These differences may have implications for behavioral outcomes, particularly under stress, as men’s tendency toward emotion suppression and risk taking is associated with greater likelihood of engaging in externalizing behaviors such as gambling in response to negative emotions (e.g., Canale et al., 2022; Sancho et al., 2019; Wong et al., 2013).

Previous studies have found that PG was strongly associated with difficulties in emotion regulation (e.g., Rogier & Velotti, 2018; Velotti et al., 2021). Emotion regulation encompasses a number of regulatory processes, including the experience of emotion as well as the underlying features of emotion, such as physiological reactivity and social, behavioral, and cognitive processes (e.g., McRae & Gross, 2020). Difficulties in emotion regulation may refer to either (1) failure to engage in regulation when needed, or (2) using a form of emotion regulation which does not match the situation (e.g., Gross & Jazaieri, 2014). Difficulties in emotion regulation is predictive of distress, maladaptive behavior, and increased risk for mental health disorders (e.g., Gross & Jazaieri, 2014, Sheppes et al., 2015).

The ability to modulate emotional responses to stress plays a critical role in how individuals cope with trauma and the extent to which they might use gambling as an escape from negative emotions (Blaszczynski & Nower, 2002; Buchanan et al., 2020; Neophytou et al., 2023; Weatherly & Cookman, 2014; Rogier & Velotti, 2018; Velotti et al., 2021). For example, findings show that increased PG was associated with exposure to stressful life events, such as injury or trauma (Luce et al., 2016; Wang et al., 2020) as well as anxiety and post-traumatic stress symptoms (e.g., Grubbs & Chapman, 2019; Lorains et al., 2011; Moore & Grubbs, 2021). Populations exposed to war related trauma, such as military veterans, are vulnerable to developing PG (e.g., Dighton et al., 2022; Etuk et al., 2020). Furthermore, several studies demonstrated that difficulties in emotion regulation and maladaptive coping partially mediated the relationship between exposure to stressful life events and heightened PG (e.g., Bergevin et al., 2006; Thurm et al., 2023). Thus, evidence supports relationships between exposure to stressful events, difficulties in emotion regulation, and PG.

However, most studies on PG rely on cross-sectional correlational designs that do not inform about the effects of trauma and stress on changes in PG over time, or about the contribution of difficulties in emotion regulation to these changes (e.g., Neophytou et al., 2023; Velotti et al., 2021). In the few longitudinal studies on changes in PG over time, data on difficulties in emotion regulation were not collected (e.g., Edgerton et al., 2015; Hagfors et al., 2023; Williams et al., 2015). Similarly, laboratory experiments that examined the effects of stress exposure on PG did not examine the potential moderating role of difficulties in emotion regulation (e.g., Kyngdon & Dickerson, 1999; Rockloff et al., 2014). To comprehensively test the theory of gambling as a coping mechanism, studies should investigate the temporal sequence by which stressful or traumatic events may lead to increased PG among those with difficulties in emotion regulation.

The goal of the current study was to address this gap in the literature by exploring the association of difficulties in emotion regulation with increased PG before and during a major collectively traumatic event. To this end, we utilized data from a longitudinal study on addiction in the Jewish Israeli general population that was collected before the mass scale terrorist attack on October 7, 2023, and during the Swords of Iron war (Human Rights Council, 2024), which constitute both existential life-threatening situations and collective trauma for many Israelis (Gutman & Landau, 2024). We hypothesized that PG will increase after the traumatic events of October 7, in comparison to before wartime. Moreover, we hypothesized that pre-post war increase in PG would be mainly noted among individuals who reported difficulties in emotion regulation problems prior to the trauma. These individuals may use gambling as a way to cope with negative emotions and “escape” the distress (e.g., Neophytou et al., 2023; Velotti et al., 2021), because of difficulties in deploying more adaptive ways of coping. Considering that Israeli men are more likely to engage in PG (e.g., Bonny-Noach, 2024; Gavriel-Fried et al., 2023a, b), we expected this effect to occur especially among men. We specifically predicted that men, who score high on self-reported measures of difficulties in emotion regulation before the traumatic events of October 7, 2023 (April 2022) would show an increase in self-reported PG from that time to after the October 7 2023 trauma and during the war (December 2023, March 2024, June 2024). We also examined if the effects are different for non-problem gamblers and people who were already at risk for problem gambling (low, moderate or problem gamblers).

## Method

### Transparency and Openness

This study was not preregistered. Data was taken from a large multi-date epidemiological project that has not yet been archived online and is available upon sensible request.

### Participants

As detailed in (Shmulewitz et al., 2024) data were collected at 4 timepoints: April 2022 (*n* = 2659), December 2023 (*n* = 4002), March 2024 (*n* = 2768) and June 2024 (*n* = 2757). Respondents were recruited from a demographically diverse Web panel (iPanel) of individuals who choose to participate in surveys for monetary compensation (~ 20 ILS for a completed survey). Participants were aged 18–70, as older individuals are less likely to participate in online surveys, and Jewish and Hebrew speaking, since substantial adaptations would be required to include different cultural groups.

In April 2022 and December 2023, the samples were constructed to match prevalence of gender, age, religiosity, education, and area of residence in the adult Jewish population in Israel (Central Bureau of Statistics, 2023), allowing deviations of up to 3% from the quotas (Fricker, 2016). The sample was not weighted. In April 2022, random panel members were invited to participate. All respondents surveyed in 2022 were invited to participate in December 2023, and those who completed the survey in December 2023 were also invited to participate in March and June of 2024. Appendix 1 in the supplementary file includes the response rate and completion rates for each timepoint.

The final sample included 899 participants who participated in all 4 timepoints (445 men and 444 women, *M*_age_ = 45.67, *SD* = 13.49). Demographic information of the sample is presented in Table 1. A sensitivity analysis using G*power (Faul et al., 2009) indicated that we could detect a small-medium effect size (*f*^*2*^ = 0.055) at 80% power in a mixed within-between subject design with 4 measurement points and 4 groups (men and women high and low in DERS).

**Table 1:** Socio-Demographic characteristics of the sample (*N* = 899; December 2023)

		*n*	Percent (%)
Gender			
Men		455	50.6%
Women		444	49.4%
Age			
18–25		75	4.0%
26–34		255	21.0%
35–49		386	21.9%
50–70		183	23.4%
Religiosity			
Secular		441	49.1%
Traditional		272	30.3%
National religious		101	11.2%
Ultra-Orthodox		85	9.5%
Residency			
Jerusalem area		95	10.6%
Tel Aviv area		125	13.9%
Central area		253	28.1%
Haifa area		205	22.8%
North area		100	11.1%
South area		100	11.1%
West Bank area		21	2.3%
Educational attainment			
High school, non-matriculation		2	0.2%
High school, matriculation		188	20.9%
Post-high school, technological		180	20.0%
Post-high school, academic		529	58.8%
Relationship status			
Married or not married couple		281	29.7%
Not married		618	71.3%
Ethnicity			
Middle Eastern/North African		327	36.4%
Ethiopian		3	0.3%
European, not Soviet Union		326	36.3%
Former Soviet Union		64	7.1%
Mixed		167	18.6%
Other		12	1.3%
Subjective socio-economic status (June 2024)			
Below average		242	26.9%
Average		367	40.8%
Above average		290	32.3%

### Procedure

After completing informed consents, participants answered demographic questions (e.g., age, gender, religiosity, geographical district, family status, education). Then, they received a battery of self-report questionnaires, measuring substance use and other addictive behaviors, psychopathology, and protective or risk factors (e.g., personal resilience, social support). Data on other measures that are not the focus of the current study are not reported here (see Appendix 2).

### Materials

#### Problem Gambling

Problem gambling was measured with the 9-item Problem Gambling Severity Index (PGSI; Ferris & Wynne, 2001) which assesses the severity of gambling problems in the past 12 months. In March and June 2024, PG was assessed for the past three months. Responses were marked on a scale ranging from 0 (*never*) to 3 (*almost always*). The overall score was calculated by summing all items (range: 0–27). The scale showed high reliability in all timepoints: Cronbach’s αs > 0.91. We also computed categories based on risk for PG (0 = ‘Non-problem gamblers’; 1–2 = ‘Low-risk gamblers’; 3–7 = ‘Moderate-risk gamblers’; and 8+ = ‘Problem gamblers; e.g., Ferris & Wynne, 2001; Gavriel-Fried et al., 2023a; cf. Currie et al., 2013).

#### Difficulties in Emotion Regulation

Difficulties in emotion regulation were measured using the 18-item Difficulties in Emotion Regulation Scale (DERS-18; Victor & Klonsky, 2016). Responses were marked on a scale ranging from 1 (*almost never*) to 5 (*almost always*). The overall score was calculated by summing all items (range: 18–90; α = 0.90), with higher scores indicating greater emotion dysregulation. We used DERS-18 scores from April 2022 (pre-war), as an individual differences variable reflecting difficulty in emotion regulation at Time 1 and predict changes in post-war measurements.

### Statistical Analysis

All analysis was conducted on IBM SPSS version 27. We began by examining the sample descriptive and zero-order correlations between the key study variables.

To test for the hypothesized interactive effects of time of measurement (1 = April 2022, 2 = December 2023, 3 = March 2024, 4 = June 2024), gender (men = 1, women = 0) and DERS measured in April 2022 (mean centered at + 1 and − 1 *SD*) on the total PGSI score, we conducted a mixed within-between general linear model analysis in two parts. First, omnibus significance testing was done in a repeated general linear model ANOVA. PGSI scores at the four timepoints were entered as a repeated variable, gender, DERS and the gender × DERS were entered as between subject variables (manually defined model). Follow up analysis were subsequently conducted using *MEMORE* model 3 (Montoya, 2019). *MEMORE* is a macro for SPSS that estimates moderation models for two-instance within-subjects/repeated measures designs following the procedures outlined by Judd et al. (2001) for testing interactions. In this study, we used *MEMORE* to compare the conditional simple slope of PGSI through time (pre-war PGSI: April 2022, and each other times: PGSI in December 2023, March 2024 and June 2024) at the different levels of gender (men or women) and DERS (at −1SD and + 1SD).

We conducted additional analyses across the different categories of PG at time 1 (2022). These analyses are exploratory because the number of participants in the PG categories was too low to test the time x gender x DERS interaction. We therefore examined this interaction only in the larger sample of the non-problem gamblers, and in the smaller risk-for-PG groups focused on the two-way interactions (using the same model).

## Results

Initial analyses indicated that men (*M* = 34.01, *SD* = 10.53) reported slightly higher DERS scores than women (*M* = 32.27, *SD* = 10.98), *t* = 2.43, *p* =.015, *Cohen’s D* = 0.16. As shown in Table 2, PGSI scores were higher for men than women, and increased over time (from April 2022 to March 2024) among men, but not among women. The distribution of the PG categories indicated that 767 participants (85.3%) were non-problem gamblers, and 14.7% were low-risk or above. See Appendix 3 for the distribution of the PG categories across gender.

**Table 2:** Means and standard errors of the problem gambling severity index according to gender and measurement time (*N* = 899)

Gender	Apr 2022 (Prior to war)	December 2023 (During war)	March 2024 (During war)	June 2024 (During war)
Men	0.87^b^ (0.14)	1.17^c^ (0.12)	1.42^d^ (0.13)	1.39^cd^ (0.15)
Women	0.52^a^ (0.14)	0.52^a^ (0.12)	0.53^a^ (0.13)	0.55^a^ (0.15)

Means with different letters were significantly different at *p* <.05

**Fig. 1:**
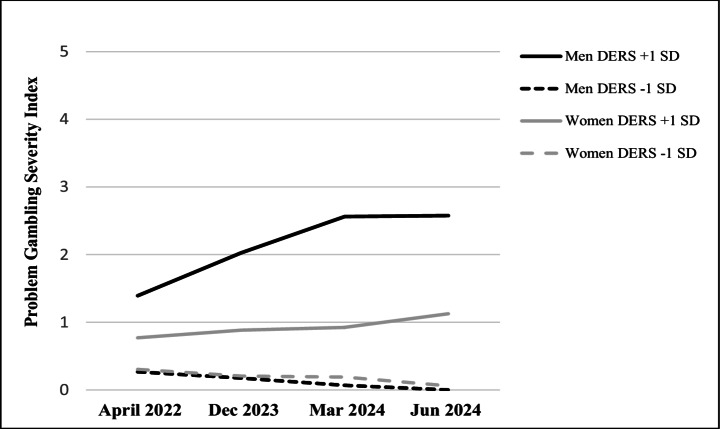
Problem gambling scores at four timepoints by gender and difficulties in emotional regulation (*N* = 899). Note. DERS = Difficulties in emotion regulation scale

In the general mixed ANOVA model of time, gender, and DERS, the following main effects were observed. First, the total PGSI score increased significantly over time *F*(1, 895) = 5.05, *p* =.025, *η*_*p*_^2^ = 0.006. Second, men showed higher total PGSI scores than women, *F*(1, 895) = 18.33, *p* <.001, *η*_*p*_^2^ = 0.020. Third, higher DERS scores predicted higher total PGSI scores, *F*(1, 895) = 104.53, *p* <.001, *η*_*p*_^2^ = 0.105. There were also two-way interactions of time × DERS, *F*(1, 895) = 19.03, *p* <.001, *η*_*p*_^2^ = 0.021, and DERS × gender, *F*(1, 895) = 22.55, *p* <.001, *η*_*p*_^2^ = 0.025, and a near-statistically significant time × gender interaction, *F*(1, 895) = 3.19, *p* =.074, *η*_*p*_^2^ = 0.004. Importantly, the time × DERS × gender three-way interaction was statistically significant, *F*(1, 895) = 4.02, *p* =.045, *η*_*p*_^2^ = 0.004, showing that changes in the total PGSI score over time differed both by gender and DERS, with the greatest increase found among men with relatively high difficulties in emotion regulation (see Fig. 1). All these results remained similar when adding age and education level as covariates (both were negatively related to PGSI and statistically significant covariates in the model, *F*s > 4.96, *p*-values < 0.027. Results were similar when using Greenhouse-Geisser correction, which corrects for type 1 error inflation due to violations of assumption of sphericity in the model.

Follow-up analyses comparing pre-war time (April 2022) to other times (December 2023, March 2024 and June 2024) indicated that for all the assessment times after the trauma of October 7 2023, the DERS × gender interaction was statistically significant, all *b*s > 0.05, *t*s > 3.73, *p*-values < 0.001. This interaction was not significant in the pre-war assessment (April 2022), *b* = 0.03, *t* = 1.73, *p* =.085. As shown in Fig. 1, simple slope comparisons indicated that a significant increase in the total PGSI score from April 2022 to each assessment time after the trauma of October 7 2023 (December 2023, March 2024, June 2024) was found only for men with relatively high DERS scores (+ 1*SD*), all *b*s > 0.53, *t*s > 3.05, *p-value*s < 0.003, but not for women (regardless of DERS scores) or men with low DERS scores (−1*SD*), all *b*s < 0.36, *t*s < 1.38, *p-value*s > 0.167. In addition, as seen in Fig. 1, high-DERS men showed a statistically significant increase in the total PGSI score from December 2023 to March 2024, *b* = 0.53, *t* = 3.63, *p* <.001, but not from March 2024 to June 2024, *b* = 0.01, *t* = 0.10, *p* =.922. Again, these changes in the total PGSI score were not significant for women (regardless of DERS scores) or men with low DERS scores, *b*s < 0.21, *t*s < 1.36, *p-value*s > 0.173.

**Fig. 2:**
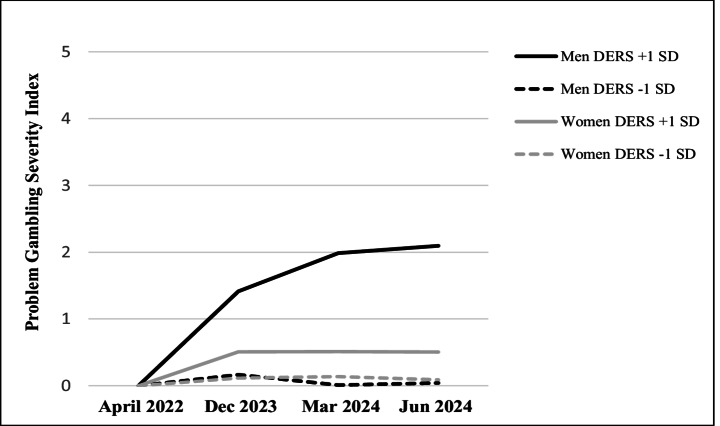
Problem gambling severity index scores at four timepoints by gender and difficulties in emotional regulation among non-problem gamblers (*n* = 767). Note. DERS = Difficulties in emotion regulation scale

**Fig. 3:**
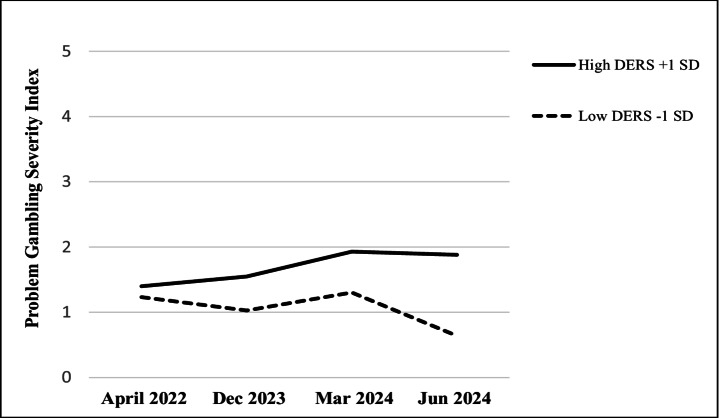
Problem gambling severity index scores at four timepoints by difficulties in emotional regulation among low-risk gamblers (*n* = 73). Note. DERS = Difficulties in emotion regulation scale

The analyses within the different PG-risk groups indicated that among non-PG participants (*n* = 767), the time × gender × DERS three-way interaction effect was statistically significant and stronger than the total sample effect, *F*(1, 763) = 19.88, *p* <.001, *η*_*p*_^2^ = 0.025 (all patterns were the same; see Fig. 2). However, different patterns emerged in other PG-risk categories. Among participants in the low-risk group (*n* = 73), the only statistically significant effect was for DERS scores (marginally statistically significant), *F*(1, 69) = 3.95, *p* =.051, *η*_*p*_^2^ = 0.054, and a time × DERS interaction, *F*(1, 895) = 4.71, *p* =.034, *η*_*p*_^2^ = 0.064. A follow-up *MEMORE* analysis indicated that low-risk PG participants with relatively high DERS scores reported higher PGSI scores than low-DERS participants in June 2024 (post-war), *b* = 0.07, *t* = 2.53, *p* =.014, but not in 2022 (pre-war), *b* = 0.01, *t* = 1.54, *p* =.128 (Fig. 3).

**Fig. 4:**
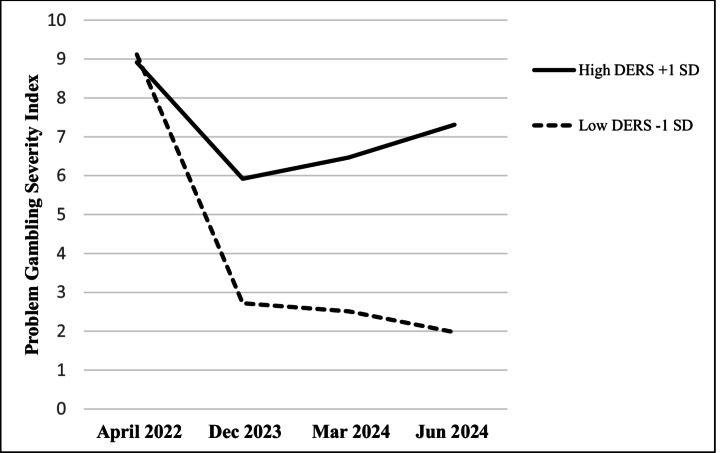
Problem gambling severity index scores at four timepoints by difficulties in emotional regulation among moderate and problem gamblers (*n* = 59). Note. DERS = Difficulties in emotion regulation scale

Among participants in the moderate-and-above risk for PG groups (*n* = 59), there was a decrease in PG over time, *F*(1, 55) = 13.79, *p* <.001, *η*_*p*_^2^ = 0.201; higher overall PGSI scores for women over men, *F*(1, 55) = 4.34, *p* =.042, *η*_*p*_^2^ = 0.073, and a time × DERS interaction, *F*(1, 55) = 5.76, *p* =.020, *η*_*p*_^2^ = 0.02. A follow-up *MEMORE* analysis indicated that in April 2022 DERS scores did not predict differences between low and high-DERS participants in PGSI, *b* = 0.01, *t* = 0.11, *p* =.915. However, from December to June 2024 low-DERS reported increasingly lower PGSI scores than high-DERS participants, *bs* > 0.13, *ts* > 2.74, *p*s < 0.009 (Fig. 4).

## Discussion

This study explored PG of Jewish Israelis adults over the course of a collectively traumatic terror attack and subsequent war. We utilized data from a longitudinal study of self-reported PG before the October 7, 2023, terror attack (April 2022) and during the Swords of Iron war (December 2023, March 2024, June 2024). Consistent with our theory and predictions, results indicated that DERS scores were associated with increased PGSI scores from before to during the war, especially among men. Additional analyses indicated that this increase in PG was predominantly evident among participants not at risk for PG before the war. Among the relatively smaller sample of participants who were categorized as moderate risk or above before the war, there was a decrease in PGSI scores among those low in difficulties in emotional regulations, independent of gender.

These findings provide a unique glimpse into the potential psychological impact of mass collective trauma on PG, while assessing PG during and after a significant national crisis—the ‘Swords of Iron’ war in Israel. They emphasize the role that preexisting difficulties in emotion regulation and gender play in moderating this response. Examining this phenomenon reveals critical insights into how different population segments respond to prolonged traumatic and life-threatening events and highlights the complexity of these effects on mental health and behaviors.

The current findings provide further support for the existing theory and research on PG and difficulties in emotion regulation (e.g., Velotti et al., 2021), the psychological function gambling-as-an-escape (e.g., Blaszczynski & Nower, 2002; Neophytou et al., 2023), and on gender differences in this domain (e.g., Merkouris et al., 2016). First, the increase in PG from the pre-war to during-war periods underscores the possible role of mass trauma in exacerbating maladaptive coping mechanisms, such as PG. This aligns with research suggesting that individuals may turn to gambling as a coping strategy during stressful and traumatic experiences that challenge existing emotional coping capacities (e.g., Luce et al., 2016; Price, 2020; Wang et al., 2020). Second, the observed rise in PG during the war among men with high DERS scores prior to the war suggests that emotional dysregulation may play a critical role in the effect (e.g., Bergevin et al., 2006; Thurm et al., 2023). Third, the gender differences among non-PG participants are consistent with prior findings, where men often report higher levels of PG compared to women (e.g., Bonny-Noach, 2024; Stark et al., 2012), as a function of difficulties in emotion regulation (e.g., Bergevin et al., 2006; Canale et al., 2022; Sancho et al., 2019).

The finding that among the small group of participants in the moderate risk and problematic gamblers categories, there was a decrease in PG, is worth further attention. It suggests that exposure to acute threat may reduce high levels of PG. This could be explained by the increased need to draw upon personal resources in order to function in times of emergency. Since PG might interfere with normal functioning, the events of the war could have forced moderate-risk and problem gamblers to change their behavior. Another explanation is that the acute threat of the October 7 attack and following war provided people with means to misattribute the anxieties and frustrations that they usually feel, onto an external threat (e.g., Ross et al., 1969; Zanna & Cooper, 1974). Considering that PG can result from distress and negative emotions (e.g., Neophytou et al., 2023; Velotti et al., 2021), for individuals who might already be suffering from problems and distress, attributing the causes of such emotions to an external threat may reduce the need to gamble as a way to cope with them. However, due to the small number of participants in the low risk for PG and above categories, additional studies in samples enriched for PG are needed to further explore this possibility.

These results underscore the potential for targeted interventions aimed at improving emotion regulation as a means to mitigate PG, especially among vulnerable populations who experienced mass trauma. The heightened vulnerability of non-PG men to PG after a mass trauma suggests possible gender differences in coping strategies and resilience resources. This may be attributed to women’s better ability to rely on social support networks or their tendency to use more adaptive coping mechanisms under stress (e.g., Merkouris et al., 2016; Chaplin et al., 2008). Hence, gender-sensitive, emotion-focused interventions in trauma-exposed populations may help reduce reliance on PG as a coping mechanism. For example, interventions that incorporate emotion regulation training, such as cognitive-behavioral therapy (CBT) and dialectical behavior therapy (DBT), could be particularly effective. Additionally, public health efforts aimed at increasing awareness and support for emotional coping strategies targeting populations exposed to war related trauma such as veterans (e.g., Dighton et al., 2022; Etuk et al., 2020; Whiting et al., 2016), particularly men with high emotion regulation difficulties, could provide a preventive buffer against the development of gambling-related problems in times of war. Although the sample only included a relatively small number of participants in the PG-risk groups, the recorded decrease in PGSI scores among those with low DERS, suggests that traumatic events may also provide some opportunity to those who are already experiencing problems with gambling to reduce their addictive behavior. This may be especially true for people with better emotion regulation capacities. However, more research is needed to further corroborate this possibility.

The current study has several important limitations to consider. First, despite the strength of the longitudinal design, without random assignment into an experimental and control condition we cannot confidently rule out alternative explanations for the results. For example, confounding variables (e.g., personality variables) may explain the association between difficulties in emotion regulation and PG or the observed pre-post war changes in PG among men. Future laboratory studies may attempt to use subtle experimental manipulation of threat (e.g., Rockloff et al., 2014) or emotional regulation of that threat (e.g., Ehring et al., 2010). Second, difficulties in emotion regulation and PG were assessed with self-report measures, but those measures are valid and widely-used. Additional measures of PG, such as observation of actual gambling behaviors, can provide a better assessment of the phenomenon. Third, the current sample was taken from the general population in Israel, however, all the participants were Israeli Jews, and Israeli Arabs who make up about 20% of Israeli population were not included. This might limit the generalizability of the findings to Israel as a whole and to other countries, cultures, and religions and emphasize the need for future studies specifically among Israeli Arabs. This is especially important, since it was previously found that being an Israeli Arab is a risk factors for PG in Israel (Gavriel-Fried et al., 2023a). Furthermore, our final sample included only participants who completed all timepoints, who may differ somewhat from the Jewish Israeli population. Moreover, this study was conducted during the Swords of Iron war, which might make the findings even more context specific. Nevertheless, the fact that the findings are congruent with previous research connecting difficulties in emotion regulation and PG in other countries (e.g., Buchanan et al., 2020; Moore, & Grubbs, 2021; Velotti et al., 2021), increases our confidence in the generalizability of the results. A fifth limitation, is that the sample included only a relatively small number of participants in the low-risk, medium-risk, or problem gamblers categories. Thus, while the current study provides important information for the general population, further research among individuals at low-risk and above for PG is needed to be more confident in those findings. Finally, in this study we did not focus on the extent to which participants’ level of trauma contributed to the effects. Further studies may also do so, by including post-traumatic stress symptoms as another within-subject factor and using more complex multi-level moderated mediational study designs.

In summary, this study highlights the significant impact of prolonged collective traumatic crisis and difficulties in emotion regulation on PG, while considering gender differences in this maladaptive behavior. These insights contribute to the understanding of the psychological responses to national crises and emphasize the need for targeted support systems for vulnerable groups, such as war veterans and men with difficulties in emotion regulation, to help mitigate the risk of PG. Future research should explore additional coping strategies and resilience resources that may mitigate the observed increase in PG during crises.

## Electronic Supplementary Material

Below is the link to the electronic supplementary material.

Supplementary Material 1 (DOCX 54.0 KB)

## References

[CR1] Allami, Y., Hodgins, D. C., Young, M., Brunelle, N., Currie, S., Dufour, M., & Nadeau, L. (2021). A meta-analysis of problem gambling risk factors in the general adult population. *Addiction*, *116*, 2968–2977. 10.1111/add.15449 33620735 10.1111/add.15449 PMC8518930

[CR2] Bergevin, T., Gupta, R., Derevensky, J., & Kaufman, F. (2006). Adolescent gambling: Understanding the role of stress and coping. *Journal of Gambling Studies*, *22*, 195–208. 10.1007/s10899-006-9010-z 16838102 10.1007/s10899-006-9010-z

[CR3] Blaszczynski, A., & Nower, L. (2002). A pathways model of problem and pathological gambling. *Addiction*, *97*, 487–499. 10.1046/j.1360-0443.2002.00015.x 12033650 10.1046/j.1360-0443.2002.00015.x

[CR4] Bonny-Noach, H. (2024). Severity of gambling behaviors: Exploring associations with venues, legality, and substance use. *Israel Journal of Health Policy Research*, *13*, 20. 10.1186/s13584-024-00604-0 38627853 10.1186/s13584-024-00604-0 PMC11020295

[CR5] Buchanan, T. W., McMullin, S. D., Baxley, C., & Weinstock, J. (2020). Stress and gambling. *Current Opinion in Behavioral Sciences*, *31*, 8–12. 10.1016/j.cobeha.2019.09.004

[CR6] Calado, F., & Griffiths, M. D. (2016). Problem gambling worldwide: An update and systematic review of empirical research (2000–2015). *Journal of Behavioral Addictions*, *5*, 592–613. 10.1556/2006.5.2016.073 27784180 10.1556/2006.5.2016.073 PMC5370365

[CR7] Canale, N., Rubaltelli, E., Calcagnì, A., Vieno, A., Giovannoni, M., Devos, G., & Billieux, J. (2022). The effects of induced sadness, stress sensitivity, negative urgency, and gender in laboratory gambling. *International Gambling Studies*, *22*, 365–389. 10.1080/14459795.2021.2002385

[CR8] Chaplin, T. M., Hong, K., Bergquist, K., & Sinha, R. (2008). Gender differences in response to emotional stress: An assessment across subjective, behavioral, and physiological domains and relations to alcohol craving. *Alcoholism, Clinical and Experimental Research,**32*, 1242–1250. 10.1111/j.1530-0277.2008.00679.x 18482163 10.1111/j.1530-0277.2008.00679.x PMC2575018

[CR9] Currie, S. R., Hodgins, D. C., & Casey, D. M. (2013). Validity of the problem gambling severity index interpretive categories. *Journal of Gambling Studies*, *29*, 311–327. 10.1007/s10899-012-9300-6 22426971 10.1007/s10899-012-9300-6

[CR10] Dighton, G., Wood, K., Armour, C., Fossey, M., Hogan, L., Kitchiner, N., & Dymond, S. (2022). Gambling problems among United Kingdom armed forces veterans: Associations with gambling motivation and posttraumatic stress disorder. *International Gambling Studies,**23*, 35–56. 10.1080/14459795.2022.2063923

[CR11] Edgerton, J. D., Melnyk, T. S., & Roberts, L. W. (2015). An exploratory study of multiple distinct gambling trajectories in emerging adults. *Journal of Youth Studies*, *18*, 743–762. 10.1080/13676261.2014.992326

[CR12] Ehring, T., Tuschen-Caffier, B., Schnülle, J., Fischer, S., & Gross, J. J. (2010). Emotion regulation and vulnerability to depression: Spontaneous versus instructed use of emotion suppression and reappraisal. *Emotion*, *10*, 563–572. 10.1037/a0019010 20677873 10.1037/a0019010

[CR13] Etuk, R., Shirk, S. D., Grubbs, J., & Kraus, S. W. (2020). Gambling problems in US military veterans. *Current Addiction Reports*, *7*, 210–228. 10.1007/s40429-020-00310-2

[CR14] Faul, F., Erdfelder, E., Buchner, A., & Lang, A. G. (2009). Statistical power analyses using G*Power 3.1: Tests for correlation and regression analyses. *Behavior Research Methods*, *41*, 1149–1160. 10.3758/BRM.41.4.1149 19897823 10.3758/BRM.41.4.1149

[CR15] Ferris, J. A., & Wynne, H. J. (2001). *The Canadian problem gambling index*. Canadian Centre on Substance Abuse.

[CR16] Fricker Jr, R. D. (2016). Sampling methods for online surveys. In N. G. Fielding, R. M. Lee, & G. Blank (Eds.), *The SAGE handbook of online research methods* (2nd ed., pp. 184–202). Sage.

[CR17] Gavriel-Fried, B., Loewenthal, A., & Vana, N. (2023a). Problem gambling severity in a nationally representative sample of the Israeli population: The moderating role of ethnonational affiliation. *Frontiers in Public Health*, *11*, 1233301. 10.3389/fpubh.2023.1233301 37799154 10.3389/fpubh.2023.1233301 PMC10548459

[CR18] Gavriel-Fried, B., Delfabbro, P., Ricijas, N., Hundric, D., D., & Derevensky, J. L. (2023b). Cross-national comparisons of the prevalence of gambling, problem gambling in young people and the role of accessibility in higher risk gambling: A study of australia, canada, Croatia and Israel. *Current Psychology: A Journal for Diverse Perspectives on Diverse Psychological Issues*, *42*, 6990–7001. 10.1007/s12144-021-02017-7

[CR19] Goodwin, B. C., Browne, M., Rockloff, M., & Rose, J. (2017). A typical problem gambler affects six others. *International Gambling Studies*, *17*, 276–289. 10.1080/14459795.2017.1331252

[CR20] Gross, J. J., & Jazaieri, H. (2014). Emotion, emotion regulation, and psychopathology: An affective science perspective. *Clinical Psychological Science*, *2*, 387–401. 10.1177/2167702614536164

[CR21] Grubbs, J. B., & Chapman, H. (2019). Predicting gambling situations: The roles of impulsivity, substance use, and post-traumatic stress. *Substance Abuse: Research and Treatment*, *13*, 1178221819852641. 10.1177/1178221819852641 31258327 10.1177/1178221819852641 PMC6591666

[CR22] Gutman, L. M., & Landau, S. D. (2024). Collective trauma and resilience for the Jewish people in the aftermath of 7th October. In R. Freedman & D. Hirsh (Eds.) *Responses to 7 October: Law and Society* (pp. 59–67). Routledge. 10.4324/9781003497417

[CR23] Hagfors, H., Vuorinen, I., Savolainen, I., & Oksanen, A. (2023). A longitudinal study of gambling motives, problem gambling and need frustration. *Addictive Behaviors*, *144*, 107733. 10.1016/j.addbeh.2023.107733 37119715 10.1016/j.addbeh.2023.107733

[CR24] Hodgins, D. C., & Stinchfield, R. (2008). Gambling disorders. In J. Hunsley, & E. J. Mash (Eds.), *A guide to assessments that work* (pp. 370–388). Oxford University Press. 10.1093/med:psych/9780195310641.003.0017

[CR25] Holdsworth, L., Hing, N., & Breen, H. (2012). Exploring women’s problem gambling: A review of the literature. *International Gambling Studies,**12*, 199–213. 10.1080/14459795.2012.656317

[CR26] Human Rights Council. (2024). Detailed findings on attacks carried out on and after 7 October 2023 in Israel. Retrieved from: https://www.ohchr.org/sites/default/files/documents/hrbodies/hrcouncil/sessions-regular/session56/a-hrc-56-crp-3.pdf

[CR27] Husky, M. M., Michel, G., Richard, J. B., Guignard, R., & Beck, F. (2015). Gender differences in the associations of gambling activities and suicidal behaviors with problem gambling in a nationally representative French sample. *Addictive Behaviors*, *45*, 45–50. 25644586 10.1016/j.addbeh.2015.01.011

[CR28] Judd, C. M., Kenny, D. A., & McClelland, G. H. (2001). Estimating and testing mediation and moderation in within-subject designs. *Psychological Methods*, *6*, 115–134. 10.1037/1082-989X.6.2.115 11411437 10.1037/1082-989x.6.2.115

[CR29] Kyngdon, A., & Dickerson, M. (1999). An experimental study of the effect of prior alcohol consumption on a simulated gambling activity. *Addiction*, *94*, 697–707. 10.1046/j.1360-0443.1999.9456977.x 10563034 10.1046/j.1360-0443.1999.9456977.x

[CR30] Lesieur, H. R., & Rosenthal, R. J. (1991). Pathological gambling: A review of the literature (prepared for the American Psychiatric Association task force on DSM-IV committee on disorders of impulse control not elsewhere classified). *Journal of Gambling Studies,**7*, 5–39. 10.1007/BF01019763 24242968 10.1007/BF01019763

[CR31] Lorains, F. K., Cowlishaw, S., & Thomas, S. A. (2011). Prevalence of comorbid disorders in problem and pathological gambling: Systematic review and meta-analysis of population surveys. *Addiction*, *106*, 490–498. 10.1111/j.1360-0443.2010.03300.x 21210880 10.1111/j.1360-0443.2010.03300.x

[CR32] Luce, C., Kairouz, S., Nadeau, L., & Monson, E. (2016). Life events and problem gambling severity: A prospective study of adult gamblers. *Psychology of Addictive Behaviors*, *30*, 922–930. 10.1037/adb0000227 27854453 10.1037/adb0000227

[CR33] Man, P. K., & Cheung, N. W. T. (2022). Do gender norms matter? General strain theory and a gendered analysis of gambling disorder among Chinese married couples. *Journal of Gambling Studies*, *38*, 123–151. 10.1007/s10899-021-10021-6 34097184 10.1007/s10899-021-10021-6

[CR34] McMillen, J., Marshall, D., Murphy, L., Lorenzen, S., & Waugh, B. (2004). *Help-seeking by problem gamblers, friends and families: A focus on gender and cultural groups*. Gambling and Racing Commission.

[CR35] McRae, K., & Gross, J. J. (2020). *Emotion Regulation Emotion*, 20, 1–9. 10.1037/emo0000703. 31961170 10.1037/emo0000703

[CR36] Merkouris, S. S., Thomas, A. C., Shandley, K. A., Rodda, S. N., Oldenhof, E., & Dowling, N. A. (2016). An update on gender differences in the characteristics associated with problem gambling: A systematic review. *Current Addiction Reports*, *3*, 254–267. 10.1007/s40429-016-0106-y

[CR37] Montoya, A. K. (2019). Moderation analysis in two-instance repeated-measures designs: Probing methods and multiple moderator models. *Behavior Research Methods,**51*, 61–82. 10.3758/s13428-018-1088-6 30306409 10.3758/s13428-018-1088-6 PMC6420436

[CR38] Moore, I. I. I., L. H., & Grubbs, J. B. (2021). Gambling disorder and comorbid PTSD: A systematic review of empirical research. *Addictive Behaviors*, *114*, 106713. 10.1016/j.addbeh.2020.106713 33268184 10.1016/j.addbeh.2020.106713

[CR39] Neophytou, K., Theodorou, M., Artemi, T. F., Theodorou, C., & Panayiotou, G. (2023). Gambling to escape: A systematic review of the relationship between avoidant emotion regulation/coping strategies and gambling severity. *Journal of Contextual Behavioral Science*, *27*, 126–142. 10.1016/j.jcbs.2023.01.004

[CR40] Nolen-Hoeksema, S. (2012). Emotion regulation and psychopathology: The role of gender. *Annual Review of Clinical Psychology,**8*, 61–87. 10.1146/annurev-clinpsy-032511-143109 10.1146/annurev-clinpsy-032511-143109 22035243

[CR41] Price, A. (2020). Online gambling in the midst of COVID-19: A nexus of mental health experience PG instead of engaged in PG concerns, substance use and financial stress. *International Journal of Mental Health and Addiction*, *20*, 1–18. 10.1007/s11469-020-00366-1 10.1007/s11469-020-00366-1 PMC7357671 32837444

[CR42] Rockloff, M. J., Browne, M., Li, E., & O’Shea, T. (2014). It’s a sure bet you’re going to die: Existential terror promotes gambling urges in problem players. *Gambling Research: Journal of the National Association for Gambling Studies (Australia)*, *26*, 33–43. Retrieved from: https://search.informit.org/doi/10.3316/informit.820214577954910

[CR43] Rogier, G., & Velotti, P. (2018). Conceptualizing gambling disorder with the process model of emotion regulation. *Journal of Behavioral Addictions*, *7*, 239–251. 10.1556/2006.7.2018.52 29936851 10.1556/2006.7.2018.52 PMC6174584

[CR44] Ross, L., Rodin, J., & Zimbardo, P. G. (1969). Toward an attribution therapy: The reduction of fear through induced cognitive-emotional misattribution. *Journal of Personality and Social Psychology*, *12*, 279–288. 10.1037/h0027800 5821854 10.1037/h0027800

[CR45] Sancho, M., De Gracia, M., Granero, R., González-Simarro, S., Sánchez, I., Fernández-Aranda, F., & Jiménez-Murcia, S. (2019). Differences in emotion regulation considering gender, age, and gambling preferences in a sample of gambling disorder patients. *Frontiers in Psychiatry,**10*, Article 625. 10.3389/fpsyt.2019.00625 31572231 10.3389/fpsyt.2019.00625 PMC6749049

[CR46] Sheppes, G., Suri, G., & Gross, J. J. (2015). Emotion regulation and psychopathology. *Annual Review of Clinical Psychology*, *11*, 379–405. 10.1146/annurev-clinpsy-032814-112739 25581242 10.1146/annurev-clinpsy-032814-112739

[CR47] Shmulewitz, D., Eliashar, R., Levitin, M. D., & Lev-Ran, S. (2023). Test characteristics of shorter versions of the alcohol, smoking and substance involvement screening test (ASSIST) for brief screening for problematic substance use in a population sample from Israel. *Substance Abuse Treatment, Prevention, and Policy,**18*, 58. 10.1186/s13011-023-00566-7 37828494 10.1186/s13011-023-00566-7 PMC10571312

[CR48] Shmulewitz, D., Levitin, M. D., Skvirsky, V., Vider, M., Eliashar, R., Mikulincer, M., & Lev-Ran, S. (2024). Comorbidity of problematic substance use and other addictive behaviors and anxiety, depression, and post-traumatic stress disorder: A network analysis. *Psychological Medicine*, *54*, 4635–4645. 10.1017/S0033291724002794 10.1017/S0033291724002794 39641244

[CR49] Stark, S., Zahlan, N., Albanese, P., & Tepperman, L. (2012). Beyond description: Understanding gender differences in problem gambling. *Journal of Behavioral Addictions*, *1*, 123–134. 10.1556/jba.1.2012.3.5 26165462 10.1556/JBA.1.2012.3.5

[CR50] Thurm, A., Satel, J., Montag, C., Griffiths, M. D., & Pontes, H. M. (2023). The relationship between gambling disorder, stressful life events, gambling-related cognitive distortions, difficulty in emotion regulation, and self-control. *Journal of Gambling Studies*, *39*, 87–101. 10.1007/s10899-022-10151-5 35921002 10.1007/s10899-022-10151-5 PMC9346051

[CR51] Tran, L. T., Wardle, H., Colledge-Frisby, S., Taylor, S., Lynch, M., Rehm, J., & Degenhardt, L. (2024). The prevalence of gambling and problematic gambling: A systematic review and meta-analysis. *The Lancet Public Health*. 10.1016/S2468-2667(24)00126-9 39025095 10.1016/S2468-2667(24)00126-9

[CR52] Velotti, P., Rogier, G., Zobel, B., S., & Billieux, J. (2021). Association between gambling disorder and emotion (dys)regulation: A systematic review and meta-analysis. *Clinical Psychology Review*, *87*, 102037. 10.1016/j.cpr.2021.102037 34022642 10.1016/j.cpr.2021.102037

[CR53] Victor, S. E., & Klonsky, E. D. (2016). Validation of a brief version of the difficulties in emotion regulation scale (DERS-18) in five samples. *Journal of Psychopathology and Behavioral Assessment*, *38*, 582–589. 10.1007/s10862-016-9547-9 10.1007/s10862-015-9514-x PMC4882111 27239096

[CR54] Wang, C., Cunningham-Erdogdu, P., Steers, M. L. N., Weinstein, A. P., & Neighbors, C. (2020). Stressful life events and gambling: The roles of coping and impulsivity among college students. *Addictive Behaviors*, *107*, 106386. 10.1016/j.addbeh.2020.106386 32272355 10.1016/j.addbeh.2020.106386 PMC8388113

[CR55] Weatherly, J. N., & Cookman, M. L. (2014). Investigating several factors potentially related to endorsing gambling as an escape. *Current Psychology,**33*, 422–433. 10.1007/s12144-014-9220-y

[CR56] Whiting, S. W., Potenza, M. N., Park, C. L., McKee, S. A., Mazure, C. M., & Hoff, R. A. (2016). Investigating veterans’ pre-, peri-, and post-deployment experiences as potential risk factors for problem gambling. *Journal of Behavioral Addictions*, *5*, 213–220. 10.1556/2006.5.2016.027 27156377 10.1556/2006.5.2016.027 PMC5387772

[CR57] Williams, R., Hann, R., Schopflocher, D., West, B., McLaughlin, P., White, N., King, K., & Flexhaug, T. (2015). Quinte longitudinal study of gambling and problem gambling. Report prepared for the Ontario Problem Gambling Research Centre. Guelph, Ontario. February 20, 2015.

[CR58] Wong, G., Zane, N., Saw, A., & Chan, A. K. K. (2013). Examining gender differences for gambling engagement and gambling problems among emerging adults. *Journal of Gambling Studies*, *29*, 171–189. 10.1007/s10899-012-9305-1 22585283 10.1007/s10899-012-9305-1 PMC4736715

[CR59] Zanna, M. P., & Cooper, J. (1974). Dissonance and the pill: An attribution approach to studying the arousal properties of dissonance. *Journal of Personality and Social Psychology*, *29*, 703–709. 10.1037/h0036651 4833431 10.1037/h0036651

