# Supplementary material for: Gambling in the Shadow of War: Evidence of Increased Problem Gambling for Men with Difficulties in Emotional Regulation During a Mass Trauma
Source: J Gambl Stud. 2025 Sep 9;42(1):219–33. doi: 10.1007/s10899-025-10417-8 (PMC13009016; doi:10.1007/s10899-025-10417-8)
Supplement: Supplementary file 1 — Supplementary Material 1 (DOCX 54.0 KB) [file 10899_2025_10417_MOESM1_ESM.docx]

**Gambling in the Shadow of War: Evidence of Increased Problem Gambling for Men with Difficulties in Emotional Regulation During a Mass Trauma**

**Supplementary Materials File**

**Appendix 1:** **Inclusion rates for the Study**

**Table S1.**

Inclusion and completion rates for the Longitudinal Study in all Timepoints.

|  | April 2022  *n* | Dec. 2023  *n* | March 2024  *n* | June 2024  *n* |
| --- | --- | --- | --- | --- |
| Were invited to participate | 11750 | 17267 | 4002 | 4002 |
| Agreed to participate | 5133 | 6765 | 3324 | 3393 |
| Were excluded due to quotas | 1944 | 1318 | - | - |
| Did not complete the survey | 505 | 1445 | 552 | 625 |
| - *failed attention checks* | *135* | *638* | *214* | *236* |
| - *dropped out* | *370* | *807* | *338* | *389* |
| Excluded based  on response patterns | 25 | - | 4 | 11 |
| Final sample | 2659 | 4002 | 2768 | 2757 |

As shown in Table S1, In April 2022, random panel members were invited to participate. Of those invited to participate (11,750), 5,133 agreed, 1,944 were excluded due to quotas, 505 did not complete the survey (135 failed attention checks, 370 dropped out), and 25 were excluded based on response patterns, for an analytical sample of 2,659. In December 2023, all respondents surveyed in 2022 were invited to participate, as were a random sample of other panel members. Of those invited to participate (17,267), 6765 agreed, 1,318 were excluded due to quotas, and 1,445 did not complete the survey (638 failed attention checks, 807 dropped out), for an analytical sample of 4,002. In March and June 2024, those who completed the survey in December 2023 were invited to participate, i.e. 4,002 participants. In March 3,324 agreed, 552 did not complete the survey (214 failed attention checks, 338 dropped out), and 4 were excluded based on response patterns, for an analytical sample of 2,768. In June 3,393 agreed, 625 did not complete the survey (236 failed attention checks, 389 dropped out), and 11 were excluded based on response patterns, for an analytical sample of 2,757.

**Appendix 2: Complete Study Procedure and Material**

**Epidemiology Study Information Manual**

Prepared by: MASKED FOR REVIEW

This document serves as a general overview of the ICA epidemiology studies and the data collected. This includes the study overview, the timepoints, and the questionnaires included, with additional details about what information was collected (i.e. what each questionnaire assesses) at which timepoints.

1. ***Study Overview***

Substance use and use disorders are leading causes of preventable morbidity and mortality worldwide, and are associated with negative social, economic, and legal consequences, as well as other mental health issues (e.g., behavioral addictions, anxiety, depression, PTSD). It is important to have up-to-date information on the prevalence of addictions in Israel, and to understand the associations between addictions and other mental health issues, as well as risk and protective factors. The first two time points (January, 2018 and April, 2022) collected data on: sociodemographics; problematic substance use; potentially addictive behaviors (electronic gaming, gambling, compulsive sexual behavior, pornography, internet use, social media use, and smartphone use); general psychopathology; treatment for mental health problems; PTSD; trauma/stressful events; social/familial support; other potential risk factors; and emotional regulation. After October 7, new data were collected (baseline: December 2023; 3 months follow-up: March 2024; 6 months follow-up: June 2024; 1 year follow- up: February 2025), to investigate the effects of October 7 and the ongoing war on addictions and mental health. Towards that goal, data was also collected on: exposure to events of October 7; ongoing stressors due to war; subjective mental health problems; changes in use before and after October 7; anxiety; depression; functional impairment; post-traumatic growth; resilience (personal, community, national); coping mechanisms; personality; and sleep disturbances.

The main study samples included general population Jewish adults (ages 18-70) living in Israel. Data were collected via iPanel, a national survey service with diverse participants. In 2018, 2022, and 2023, the samples were quasi-representative, i.e., prevalences of key sociodemographics (gender, age, religiosity, and area of residence [2023]; also education in 2018 and 2022) matched prevalence in the population, according to the Israel Central Bureau of Statistics. Each time point after 2018 tried to re-survey as many as previous respondents as possible. In 2024 (March and June), only respondents from 2023 were re-surveyed.

1. ***Time points***

Table 1 shows the number of respondents at each time point, how many respondents were new, and how many had been interviewed at previous timepoints.

**Table 1. Respondents at each time point**

|  | *2018* | *2022* | *2023* | *March 2024* | *June 2024* |
| --- | --- | --- | --- | --- | --- |
| Collection dates |  | *Apr 24-May 10* | *Nov 26-Dec 12* | *Mar 6-31* | *June 17-July 25* |
| *2018* | **4019** | 1176 | 760 | 625 | 619 |
| *2022* |  | **2659** | 1343 | 1053 | 1052 |
| *2023* |  |  | **4002** | 2768 | 2757 |
| *2024 March* |  |  |  | **2768** | 2270 |
| *2024 June* |  |  |  |  | **2757** |

- From the April 2022 sample (2659), 1,182 were also surveyed in 2018.
- From the December 2023 sample (4002), 1,343 were also surveyed in 2022; 690 were surveyed at all 3 time points; and 72 were surveyed in 2018 but not April 2022.
- From the March 2024 sample (2768), all were surveyed in 2023; 1,053 were also surveyed in 2022; 570 were surveyed at all 4 time points; and 57 were surveyed in 2018 but not April 2022.
- From the June 2024 sample (2757), all were surveyed in 2023; 2270 were surveyed in March 2024; 1,052 were also surveyed in 2022; 494 were surveyed at all 5 time points; and 127 were surveyed in 2018 but not April 2022.
- 899 were surveyed in 2022, 2023, and both time points in 2024
- 542 were surveyed in 2018, 2023, and both time points in 2024

*Information about the survey response rate*:

**2022**: Of those invited to participate (11,750), 4,948 agreed, 1,944 were excluded due to quotas,

505 did not complete the survey (135 failed attention checks, 370 dropped out), and 25 were excluded based on response patterns (duplicates), for an analytical sample of 2,474.

**2023:** Of those invited to participate (17,267), 6765 agreed, 1318 were excluded due to quotas, and 1445 did not complete the survey (638 failed attention checks, 807 dropped out), for an analytical

sample of 4002.

**March 2024**: Of those invited to participate (4002), 3324 agreed, 552 did not complete the survey (214 failed attention checks, 338 dropped out), and 4 were excluded based on response patterns (duplicates), for an analytical sample of 2768.

**June 2024**: Of those invited to participate (4002), 3393 agreed, 625 did not complete the survey (236 failed attention checks, 389 dropped out), and 11 were excluded based on response patterns (duplicates), for an analytical sample of 2757.

1. ***Questionnaires Included in Dataset***
2. **Sociodemographics** – in-house questions, about age, gender, area of residence, religiosity, education, ethnicity, marital status, work status, children, economic status, and army service; immigrant status (2018, 2022); HMO (2022); physical health (2023 and on), sexual identity (2023); work/school change since Oct 7 (2023). March and June 2024 included a shorter set of sociodemographics that could have changed from December 2023.
3. **Substances**

**ASSIST 3.1**

Description for 2023, 2024: The widely used, reliable, and valid Alcohol, Smoking and Substance Involvement Screening Test (ASSIST 3.1) was administered to assess problematic substance use^1,2^. Respondents selected substances they ever used non-medically (tobacco, alcohol, cannabis, sedatives, prescription stimulants, prescription opioid painkillers), and then answered six questions related to the frequency of use, craving, and consequences of use, for each substance ever used. Responses were weighted and summed into substance involvement scores. Symptoms that were not assessed due to logical skips, e.g., symptoms for a substance that was never used, were coded as 0 (no/never). A binary variable for problematic use was defined as moderate (score of ≥11 for alcohol, ≥4 for other substances) or high (≥27) risk levels.

These cut-offs were shown to have concurrent and discriminant validity, and are considered parallel to substance abuse (moderate) and dependence (high)^1,2^.

Differences from previous surveys: Craving was asked to everyone with lifetime use, not only those with past three months use; in 2022, prescription stimulants and prescription opioid painkillers were asked in a separate module, and were not asked at all in 2018; 2018 and 2022 asked specifically about cocaine, amphetamines, hallucinogens, inhalants, opiates (heroin, morphine, codeine, etc.); these were included in the "other" category in 2023/24/25; 2018 and 2022 wrote "sedative drugs" (changed to "prescription sedatives" in 2023/24).

1. **Gambling – PGSI**

The Problem Gambling Severity Index (PGSI)^3^, which is widely used, with good reliability and validity^4^, assessed problematic gambling in the past 12 months. The PGSI includes nine items assessing the frequency of gambling behaviors, with four response options: (0) never; (1) sometimes; (2) most of the time; and (3) almost always. The overall score was calculated by summing all 9 items (range: 0-27), and a binary variable indicating problematic gambling was created based on a score of ≥5, the cut-off that led to the best discriminant validity and definition of gambling problems^4,5^.

1. **Compulsive sexual behavior – BYSAS**

The Bergen-Yale Sex Addiction Scale (BYSAS), a valid measure similar to other widely used measures of behavioral addiction, assessed current problematic compulsive sexual behavior^6^. The BYSAS includes 6 items assessing frequency of sexual behaviors, with 5 response options: (0) very rarely; (1) rarely; (2) sometimes; (3) often; (4) very often. The overall score was calculated by summing all 6 items (range: 0-24), and a binary variable indicating problematic compulsive sexual behavior was positive if a response of "often" or higher was endorsed for more than half (≥4) of the items.

1. **Pornography – PPUS**

The Problematic Pornography Use Scale (PPUS)^7^, which showed good reliability and validity, was used to assess problematic pornography use within the past year. The PPUS includes 12 items assessing statements about pornography use, with 6 response options: (0) never true; (1) rarely true; (2) sometimes true; (3) often true; (4) very often true; (5) almost always true. The overall score was calculated by summing all 12 items (range: 0-60), and a binary variable indicating problematic pornography use was positive if a response of "often true" or higher was endorsed for more than half (≥7) of the items, similar to the definition of binary GAS^6,8^.

1. **Paying for sex**

Four questions on payment for sex developed in-house, based on reviewing relevant literature including the recommendation to ask about frequency^9^. Payment for sex was for one of the following services: escort institutes, discreet apartments, “health”\massage institutions, strip clubs or services, escort services, street prostitution, and webcams or cybersex. Question 1 was about ever paying for sex, with 4 response options: (1) no; (2) yes, but not in the last year; (3) yes, in the last year; (4) prefer not to say. Question 2 was asked to respondents who ever paid for sex, and asked about the number of times they ever paid for sex, with 4 response options: (1) once; (2) 2-5 times; (3) more than 5 times; (4) prefer not to say. Those who responded 2 or more times were asked about the frequency of paying for sex in the last year, with 7 response options: (1) once or twice; (2) a few times, more than once or twice; (3) every month; (4) a few times a month; (5) every week; (6) every day or almost every day; (7) prefer not to say. Question 4 asked about change in paying for sex before October 7^th^ in comparison to after October 7^th^, and had 6 response options: (1) Did not before October 7^th^, nor after; (2) Did not before October 7^th^, and started after; (3) Did before October 7^th^ and there was an increase after; (4) Did before October 7^th^ and there was a decrease, or stopped completely, after; (5) Did before October 7^th^ and there was no change after; (6) prefer not to say. Questions were shorter/fewer in June 2024.

1. **Gaming – GAS**

The Game Addiction Scale (GAS)^8^, an established measure with good psychometric properties^10^, assessed problematic electronic gaming in the past 6 months. The GAS includes 7 items assessing the frequency of gaming behaviors, with five response options: (1) never; (2) rarely; (3) sometimes; (4) often; (5) very often. The overall score was calculated by summing all items (range: 7-35), and a binary variable indicating problematic gaming was positive if a response of "often" or higher was endorsed for more than half (≥4) of the items, as suggested previously, similar to scoring for other behavioral addiction measures^6^. Although a number of cut-offs for problematic gaming were suggested by the authors of the GAS^8^, we selected this definition, since endorsing a behavior "often" or more would be frequent enough to qualify as problematic.

1. **Internet – IAT**

The Internet Addiction Test (IAT)^11^, a valid, reliable, and widely used measure worldwide^12^, assessed problematic internet use in the past month. The IAT includes 20 items assessing frequency of internet use behaviors, with 6 response options: (0) not relevant; (1) rarely; (2) sometimes; (3) often; (4) very often; (5) always. The overall score was calculated by summing all 20 items (range: 0-100), and a binary variable indicating problematic internet use was positive for scores ≥50, as in^13^.

Included in 2018, 2022, 2023, 2024,

1. **Social media**

**BSMAS**

The Bergen Social Media Addiction Scale (BSMAS)^14^, a valid and widely used measure^15^, assessed problematic social media use in the past 12 months. The BSMAS includes 6 items assessing frequency of social media behaviors, with 5 response options: (1) very rarely; (2) rarely; (3) sometimes; (4) often; (5) very often. The overall score was calculated by summing all 6 items (range: 6-30), and a binary variable indicating problematic social media use was positive if a response of "often" or higher was endorsed for more than half (≥4) of the items, as for other behavioral addictions^6^.

Included in 2018, 2022, 2023, 2024

1. **Smartphone – SAS-SV**

The Smartphone Addiction Scale, short version (SAS-SV)^16^, a reliable, valid and commonly used measure^17^, assessed current problematic smartphone use. The SAS-SV includes 10 items assessing degree of agreement with statement about smartphone use, with 6 response options ranging from very strongly disagree (1) to very strongly agree (6). The overall score was calculated by summing all 10 items (range: 10-60), and a binary variable indicating problematic smartphone use was positive for scores ≥31 for men or ≥33 for women.

Included in 2023, 2024

1. **Subjective – addiction and mental health problems**

Eighteen in-house questions assessed experiencing perceived problems due to use of substances (tobacco, alcohol, cannabis, prescription sedatives, prescription stimulants, prescription opioid painkillers, illegal drugs) or potentially addictive behaviors (gaming, gambling, compulsive sexual behavior, pornography, and social media, internet, smartphone use), and mental health problems (anxiety, depression, PTSD, anxiety or other mood disorder), prior to October 7. Each was assessed separately with 3 response options: (1) during the year prior to October 7 (2) lifetime, until the year prior to October 7 (3) had never experienced.

Included in 2023

1. **Change in use, before/after Oct 7**

The 2023 survey included 14 in-house questions assessing perceived changes in the amount or frequency of substance (tobacco, alcohol, cannabis, prescription sedatives, prescription stimulants, prescription opioid painkillers, illegal drugs) and potentially addictive behaviors (gaming, gambling, compulsive sexual behavior, pornography, and social media, internet, smartphone use) use after October 7. Each was assessed separately with 4 response options: (1) no change (2) yes, there was an increase (3) yes, there was a decrease (4) not relevant. These responses were changed in the 2024 survey because we realized that there was not enough information about who was or was not using; new response options were: (1) neither before nor after (2) didn’t use before, and started after (3) yes used before, and there was an increase in the amount\frequency of use after (4) yes used before, and there was a decrease in the amount\frequency of use (5) yes used before, and there was no change after. Additionally, in 2024, perceived change in use were assessed for the month after October 7 relative to the month before.

Included in 2023, 2024 (March)

1. **Treatment**

The 2022 survey included extensive questions on treatment, adapted from the US National Survey on Drug Use and Health^18^. Problematic substance use, potentially addictive behaviors, and other mental health issues were assessed separately. Questions included self-report need for treatment, if received treatment, if it helped, and why did not receive treatment. The 2023 survey included 2 in-house questions, one about whether treatment was received for: (1) substances; (2) behaviors; (3) stress symptoms; (4) anxiety/mood disorders; and (5) other issues; and the other question asked about treatment after October 7 relative to before.

1. **PTSD – PCL**

The Posttraumatic Stress Disorder Checklist – DSM-5 version (PCL-5)^19,20^, a reliable and valid widely used screening tool^21^, was used to assess past month PTSD symptoms. The PCL-5 includes 20 items assessing how much respondent was bothered by PTSD-related problems, with 5 response options: (0) not at all; (1) a little bit (2) moderately; (3) quite a bit; (4) extremely. The overall score was calculated by summing all 20 items (range: 0-80). Initially, a binary variable indicating potential PTSD was positive for scores ≥33; in November 2024 we decided to use a DSM-5 based definition.

In 2023/24/25, PCL was asked specifically due to the events of October 7^th^ and the ongoing war; in 2018, asked about PTSD from the worst event from the previous questionnaire (LEC) and in 2022, asked about PTSD from any possibly traumatic event may have ever experienced.

In 2023 and 2024 an additional item related to acute stress was included, as well as questions about when PCL symptoms began and duration of symptoms.

Included in 2018, 2022, 2023, 2024

1. **General psychopathology – BSI**

In 2022 and 2018, general psychopathology was assessed using the Brief Symptom Inventory (BSI), which contains 53 items assessing 9 inter-related domains: anxiety; depression; hostility; interpersonal sensitivity; obsession-compulsion; paranoid ideation; phobic anxiety; psychoticism; and somatization^22,23^. Each item assessed how much the respondent was distressed or bothered by a specific problem in the past month, with 5 response options: (0) not at all; (1) a little bit; (2) moderately; (3) quite a bit; (4) extremely. The BSI items show good internal consistency and are useful indicators of general psychopathology^24^. The overall score, called the general severity index (GSI), was calculated from the mean values for all 53 items, standardized by transformation to a T-score (mean=50, SD=10), using sample norms, by gender (from 2022). In 2018, a sum score was created.

In 2023 and 2024, a shorter, 10 item version was included, the SCL-10R^25^, and a sum score was created. For 2018 and 2022, the shorter measures were created as well to match later datasets.

Included in 2018, 2022, 2023, 2024

1. **Anxiety – GAD-7**

The General Anxiety Disorder 7 (GAD-7)^26^, a widely used valid and reliable screening tool, was used to assess general anxiety symptoms over the past two weeks. The GAD-7 includes 7 items assessing how many days respondent was bothered by anxiety-related problems, with 4 response options: (0) not at all; (1) several days; (2) more than half the days; (3) nearly every day. The overall score was calculated by summing all 7 items (range: 0-21), and a binary variable indicating potential anxiety was positive for scores ≥10.

Included in 2023, 2024

1. **Depression – PHQ-9**

The Patient Health Questionnaire 9 (PHQ-9)^27^, a widely used, valid and reliable screening tool, was used to assess depression symptoms over the past two weeks. The PHQ-9 includes 9 items assessing how many days respondent was bothered by depression-related problems, with 4 response options: (0) not at all; (1) several days; (2) more than half the days; (3) nearly every day. The overall score was calculated by summing all 9 items (range: 0-27), and a binary variable indicating potential depression was positive for scores ≥15.

Included in 2023, 2024

1. **Functional impairment – SDS**

The Sheehan Disability Scale (SDS)^28^, a widely used, reliable tool, was used to assess how much symptoms of stress and mental health issues disrupted work, social life, or family life in the past week. The SDS include 3 items with 5 response options: (0) not at all; (1) mildly; (2) moderately; (3) markedly; (4) severely. These responses were adapted from the original SDS that had 10 response options which were categorized into those 5 categories. The overall score was calculated by summing all 3 items (range: 0-12).

Included in 2023, 2024

1. **Post-traumatic growth – PTGI**

The Post Traumatic Growth Inventory (PTGI)^29^ is the most commonly used inventory^30^ to measure personal growth, and comprises 5 factors: personal strength, relating to others, new possibilities, spiritual change, and appreciation of life. The PTGI includes 21 items that relate to changes experienced in the aftermath of a highly stressful or traumatic life event. Here, respondents rated the degree to which the various changes had occurred since the war began, with 6 response options: (0) not at all; (1) a very small degree; (2) a small degree; (3) a moderate degree; (4) a great degree; (5) a very great degree. The PTGI can be considered as a single general factor, or as 5 factors^29^. The overall score was calculated by summing all 21 items (range: 0-105), with higher scores indicative of greater growth.

Included in 2023, 2024 (March)

1. **Adverse childhood experiences – WHO ACE-IQ**

Traumatic experiences during childhood (before age 18) were assessed using questions adapted from the World Health Organization's Adverse Childhood Experiences – International Questionnaire (ACE-IQ)^31^. 6 questions asked about how often respondent experienced (1) emotional abuse; (2) physical abuse; (3, 4) sexual abuse; (5) emotional neglect; (6) physical neglect. Response options included: (1) never; (2) once; (3) a few times; (4) many times. The emotional neglect item had a mistake so should not be used. An additional 5 questions asked about whether someone in the home had (1) problems with drinking or drugs; (2) problems with depression, mental health, or suicidality; (3) been in jail; (4) been abused; or (5) if parents got divorced or died. Response options were (1) yes; (2) no.

Included in 2023

1. **October 7 exposure**

The 2023 survey included 4 in-house items about the manner and extent of exposure to the traumatic events of October 7. There were 4 response options, and it was possible to choose more than one answer: (1) Happened to me; (2) Happened to a close family member; (3) Happened to someone I know; (4) none of the above. The first item was about being in an attacked area in the south on October 7^th^. The second item was about exposure during duty in the security forces. The third item was about being in a place with missiles. The fourth item was about severely injured or death due to the events of October 7. The appropriate variables will be created based on the research question.

Included in 2023

1. **Ongoing stressors**

A number of in-house questions about ongoing stressors related to the war were asked. The 2023 survey included 16 items about: refugee status, changes in work, children at home, army/security service, and frequency of ongoing exposure to war/missiles, feelings of danger, financial insecurity, exposure to uncensored content and hate speech on social media.

The 2024 survey included an additional 10 items, to provide more details about army/security service and refugee status, frequency of stress regarding academic or occupational future, and in the relationship with children or partner, and about duration of exposure to social media or news.

1. **Social support – MSPSS**

The Multidimensional Scale of Perceived Social Support (MSPSS)^32^ is a widely used, highly reliable and valid tool^33^ used to assess subjects perceived level of social support from three social circles (significant other, family, friends). The MSPSS includes 12 items about how strongly respondents agree/disagree with statements about their perceived level of social support with 7 response options: (1) very strongly disagree; (2) strongly disagree; (3) mildly disagree; (4) neutral; (5) mildly agree; (6) strongly agree; (7) very strongly agree. The overall score was calculated by summing all 12 items (range: 12-84), with higher scores indicating greater social support.

Included in 2018 (~half the sample), 2022, 2023, 2024 (March).

1. **Personal resilience – CD-RISC**

The Connor–Davidson Resilience Scale (CD-RISC)^34^ is a widely used, highly reliable tool to assess resilience levels. The CD-RISC includes 10 items about how often statements regarding resilience are true, with 5 response options: (0) not true at all; (1) infrequently true; (2) sometimes true; (3) frequently true; (4) true nearly all the time. The overall score was calculated by summing all 10 items (range: 0-40), with higher scores indicating greater resilience.

Included in 2023, 2024

1. **Emotional regulation – DERS**

The Difficulties in Emotion Regulation Scale - 18 (DERS-18)^35^ is a widely used and valid assessment tool designed to measure emotion dysregulation. The DERS-18 includes 18 items grouped into six subscales, each addressing a specific aspect of emotion regulation: awareness, clarity, acceptance, strategies, goals, and impulse control. Respondents rate how often statements are true about them, with 5 response options: (1) almost never; (2) sometimes; (3) about half the time; (4) most of the time; (5) almost always. The overall score is calculated by summing all 18 items (range: 18-90), with higher scores indicating greater emotion dysregulation.

Included in 2018 (longer version, on ~ half the sample; also created shorter version), 2022, 2023, 2024.

1. **Personality – BFI**

Big Five Inventory- 10 (BFI-10)^36^ is a shortened version of the highly reliable and widely used BFI-44. The BFI-10 includes 10 items addressing 5 distinct personality traits; extraversion, agreeableness, conscientiousness, neuroticism, and openness to experience. Respondents rate how much they agree with statements about themselves, with 5 response options: (1) does not agree at all; (2) disagree; (3) does not disagree or agree; (4) agree; (5) strongly agrees. Pairs of items were summed to create 5 traits (but alphas were poor).

Included in 2018 (longer version, on ~ half the sample), 2024

1. **Community resilience – CCRAM10**

The Conjoint Community Resiliency Assessment Measure (CCRAM-10)^37^ assesses perceived community resilience. The CCRM-10 includes 10 items about how much respondent agrees with statements about their community, with 5 response options: (1) does not agree at all; (2) mildly agrees; (3) moderately agrees; (4) strongly agrees; (5) very strongly agrees. The overall score is calculated by summing all 10 items (range: 10-50), with higher scores indicating perception of greater community resilience.

Included in 2023, 2024

1. **National resilience**

The National resilience (NR) scale^38^ assesses perceived national resilience. The NR-13 includes 10 items assessing identification with my country, solidarity and social justice, and 3 items assessing trust in public institutions. Respondents stated how much they agree with the first 10 statements, with response options from 1 (very strongly disagree) to 6 (very strongly agree). For the last 3 items, respondents rated how much they trust public institutions, with response options from 1 (does not trust at all) to 6 (trusts very strongly).

Included in 2023, 2024 (June)- 16 item version^39^**.**

1. **Coping mechanism – COPE**

The Brief COPE inventory was used to assess coping strategies with stress in everyday life^40^. The original inventory consists of 14 factors each composed of 2 items, for 28 items in total. To conserve space, from each of 10 factors we removed 1 item that appeared quite redundant with the other item in that factor, for a total of 18 items. Frequency of use of each coping strategy was assessed with 4 response options: (0) never; (1) a small extent; (2) a moderate extent; (3) a very large extent.

Included in 2024 (March)

1. **Impulsivity -BIS-Brief**

The Barratt Impulsiveness Scale-Brief (BIS-Brief)^41^, a widely used, valid and reliable screening tool, was used to assess impulsivity. The BIS-Brief includes 8 items assessing how respondent usually behaves or thinks, with 4 response options: (1) never/rarely; (2) occasionally; (3) often; (4) almost always/always. The overall score was calculated by summing all 8 items (range: 8-32).

Included in 2024

1. **Alcohol – AUDIT**

The Alcohol Use Disorders Identification Test (AUDIT)^42^, a widely used, valid and reliable screening tool, was used to assess problematic alcohol use in the past year. The AUDIT includes 10 items assessing alcohol use behaviors, starting with (1) frequency of use, with 5 response options: (0) Never; (1) Monthly or less; (2) 2-4 times a month; (3) 2-S3 times a week; (4) 4 or more times a week. Those who answered at least monthly or less were then asked about (2) number of drinks per drinking day, with 5 response options: (0) 1 or 2; (1) 3 or 4; (2) 5 or 6; (3) 7, 8, or 9; (4) 10 or more; and (3-8) frequency of drinking behavior or consequences, with 5 response options: (0) Never; (1) Less than monthly; (2) Monthly; (3) Weekly; (4) Daily or almost daily. All respondents were asked two final questions about injuring someone due to drinking and others concerned about respondent's use, with 3 response options: (0) no; (2) yes, but not in last year; (4) yes, in last year. The overall score was calculated by summing all 10 items (range: 0-40), and a binary variable of potentially problematic use was positive for scores ≥8.

Included in 2018, 2022.

1. **Drugs - DAST-10**

The Drug Abuse Screening Test (DAST-10), a widely used, valid and reliable screening tool^43^, was used to assess problematic use of any drug in the past year. The DAST-10 includes 10 items assessing drug use behaviors, with 2 response options: (0) no; (1) yes, except for item 3 which is reversed (0=yes, 1=no). The overall score was calculated by summing all 10 items (range: 0-10), and a binary variable of potentially problematic use was positive for scores ≥3.

Included in 2018, 2022

1. **Compulsive sexual behavior – HBI**

Current problematic compulsive sexual behavior was assessed using the Hypersexual Behavior Inventory (HBI), which showed good psychometric properties^44,45^. The HBI includes 19 items assessing frequency of sexual behaviors, with 5 response options: (1) never; (2) rarely; (3) sometimes; (4) often; (5) very often. The overall score was generated by summing all 19 items (range: 19-95), and a binary variable indicating problematic sexual behavior was created based on a score of 53 or more, as in^13,46^, which showed good validity and is considered clinically significant^44^.

Included in 2018, 2022

1. **Attachment – ECR**

Attachment style was assessed using the Experiences in Close Relationships Scale (ECR-12)^47^. The scale consists of 12 items, 6 tapping attachment anxiety (e.g., “I worry about being abandoned”), and 6 tapping attachment avoidance (e.g., “I feel comfortable depending on others )reverse coding)). Responses were marked on a scale ranging from 1 (strongly disagree) to 7 (strongly agree). Mean (total) scores were computed for each participant on each subscale, with higher scores reflecting greater anxiety and avoidance.

Included in 2018 on ~half of the respondents (longer version ECR-36 and created shorter version) and in June 2024.

1. **List of threatening experiences – LTE**

The occurrence of potentially traumatic (threatening) experiences in the past 12 months was assessed using the List of Threatening Experiences (LTE) ^58,59^, which includes 12 experiences in the following categories: serious illness/injury to yourself, close family member; parent, child, partner, family member died; separated from partner; serious interpersonal problems; work/financial problems; legal problems; something important was lost or stolen.

Included in 2018, 2022

1. **Early childhood- family economics – ELS**

Childhood socioeconomic status and family environment were assessed with 11 in-house items, adapted in part from xx^31^. Items included questions about family having enough money, parents jobs, household environment (positive/warm; arguments, physical/verbal abuse), community stability, and household members experience of problematic substance use, mental health disorders. For each item, respondent was asked how true it was for them, until age 10; with the following response options: 1=very much don't agree; 2=don't agree; 3=somewhat don't agree; 4=neutral; 5=somewhat agree; 6=agree; 7=very much agree.

Included in 2018 (not in 4035 dataset, yes in larger?), 2022 (added family history of mental health issues)

1. **Gambling – NODS**

Problematic gambling was assessed with the National Opinion Research Center Diagnostic Screen for Gambling Problems (NODS)^60^. The NODS includes 10 yes/no questions about lifetime gambling behaviors, with the total score ranging from 0-10. Scores of 0 are considered "no problems"; 1-2, "low risk"; 3-4, "moderate risk"; and 5-10, "severe risk".

Included in 2018, 2022

1. **Pornography – PPCS**

Problematic use of pornography was assessed using the Problematic Pornography Consumption Scale (PPCS)^61^, which includes frequency of 6 behaviors over the past 6 months, with the following response options: (1) never; (2) infrequently; (3) occasionally; (4) sometimes; (5) frequently; (6) very frequently; (7) all the time. Total score ranged from 6-42, with values 20 or above considered "problematic".

Included in 2022

1. **Online activities– Olact**

In house, assessing how much respondent's free time was spent on a regular day (not weekend) in the past month on the following online activities: social media; watching videos; dating sites; adult entertainment sites; work email; chatting; discussion groups; news; auction sites; gambling; gaming; shopping; personal email; stock trading; just browsing; other; responses included: (1) never; (2) less than an hour; (3) 1-2 hours; (4) 2-3 hours; (5) 3-5 hours; (6) more than 6 hours.

Included in 2018 (not in 4035 dataset, we might be able to find the data if someone is really interested), 2022

1. **Smartphone – SCT**

Problematic smartphone use was assessed using the Smartphone Compulsion Test^62,63^, which includes 15 yes/no questions about smartphone use. Total scores ranged from 0-15, with scores 9 or above considered "problematic".

Included in 2018 (not in 4035 dataset, yes in larger), 2022

1. **Cell phone activities – Cell**

In house, assessing how much time is spent on a regular day on the following cell phone activities: browsing the internet, email, Facebook, WhatsApp, Instagram, twitter, other; responses include: 1=not at all; 2=up to 15 minutes; 3=15-30 minutes; 4=30-60 minutes; 5=1-2 hours; 6=2-3 hours; 7=more than 3 hours.

Included in 2018 (not in 4035 dataset, we might be able to find the data if someone is really interested), 2022

These were assessed in 2018 but have not been processed.

1. **Stressful events – LEC**

The Life Events Checklist (LEC)^64,65^ was used to assess potentially traumatic experiences.

Included in 2018

1. **Early childhood stability – UP, also 2 abuse items**

Included in 2018

1. **Sensation seeking – BSSS**

The Brief Sensation Seeking Scale (BSSS)^66^ assessed sensation-seeking behavior.

Included in 2018 on ~half of the respondents

1. **Personality – SD3**

The Short Dark Triad (SD3)^67^ was used to assess Machiavellianism, narcissism, and psychopathy.

Included in 2018 on ~half of the respondents

1. **Couple relationship – DAS-7**

The Dyadic Adjustment Scale (7 item version)^68^ was used to assess relationships.

Included in 2018 on ~three quarters of the respondents

1. **Loneliness – UCLA**

The UCLA Loneliness Scale (Version 3)^69^ was used to assess loneliness and isolation.

Included in 2018 on ~half of the respondents

1. **Locus of control – MLOC**

The Multidimensional Locus of Control Scale (MLOC)^70^ was used to assess beliefs about locus of control.

Included in 2018 (not in 4035 dataset, we might be able to find the data if someone is really interested)

**References**

1. Humeniuk, R., Henry-Edwards, S., Ali, R., Poznyak, V. & Monteiro, M. *The Alcohol, Smoking and Substance Involvement Screening Test (ASSIST): Manual for Use in Primary Care.* . (World Health Organization, Geneva, 2010).

2. Humeniuk, R. *et al.* Validation of the alcohol, smoking and substance involvement screening test (ASSIST). *Addiction* 103, 1039–1047 (2008).

3. Stinchfield, R., Govoni, R. & Frisch, G. R. A review of screening and assessment instruments for problem and pathological gambling. . in *Research and measurement issues in gambling studies* (eds. Smith, G., Hodgins, D. C. & Williams, R. J.) 179–213 (Academic Press, Burlington, MA, 2007).

4. Currie, S. R., Hodgins, D. C. & Casey, D. M. Validity of the Problem Gambling Severity Index interpretive categories. *J Gambl Stud* 29, 311–27 (2013).

5. Williams, R. J. *et al.* Gambling and Problem Gambling in Canada in 2018: Prevalence and Changes Since 2002. *The Canadian Journal of Psychiatry* 66, 485–494 (2021).

6. Andreassen, C. S., Pallesen, S., Griffiths, M. D., Torsheim, T. & Sinha, R. The Development and Validation of the Bergen-Yale Sex Addiction Scale With a Large National Sample. *Front Psychol* 9, 144 (2018).

7. Kor, A. *et al.* Psychometric development of the Problematic Pornography Use Scale. *Addictive behaviors* 39, 861–8 (2014).

8. Lemmens, J. S., Valkenburg, P. M. & Peter, J. Development and Validation of a Game Addiction Scale for Adolescents. *Media Psychol* 12, 77–95 (2009).

9. Shilo, G., Gewirtz-Meydan, A. & Peled, E. Men Who Pay for Sex Once, More than Once, or Not at All: The Associations between Attitudes toward Paying for Sex, Socio-Demographic Characteristics, and Frequency of Sex Payment. *J Sex Res* 58, 754–762 (2021).

10. King, D. L. *et al.* Screening and assessment tools for gaming disorder: A comprehensive systematic review. *Clin Psychol Rev* 77, 101831 (2020).

11. YOUNG, K. S. Internet Addiction: The Emergence of a New Clinical Disorder. *CyberPsychology & Behavior* 1, 237–244 (1998).

12. Pawlikowski, M., Altstötter-Gleich, C. & Brand, M. Validation and psychometric properties of a short version of Young’s Internet Addiction Test. *Comput Human Behav* 29, 1212–1223 (2013).

13. Sela, Y., Bar-Or, R. L., Kor, A. & Lev-Ran, S. The Internet addiction test: Psychometric properties, socio-demographic risk factors and addictive co-morbidities in a large adult sample. *Addictive behaviors* 122, 107023 (2021).

14. Andreassen, C. S. *et al.* The relationship between addictive use of social media and video games and symptoms of psychiatric disorders: A large-scale cross-sectional study. *Psychology of Addictive Behaviors* 30, 252–262 (2016).

15. Casale, S., Akbari, M., Seydavi, M., Bocci Benucci, S. & Fioravanti, G. Has the prevalence of problematic social media use increased over the past seven years and since the start of the COVID-19 pandemic? A meta-analysis of the studies published since the development of the Bergen social media addiction scale. *Addictive behaviors* 147, 107838 (2023).

16. Kwon, M., Kim, D.-J., Cho, H. & Yang, S. The Smartphone Addiction Scale: Development and Validation of a Short Version for Adolescents. *PLoS One* 8, e83558 (2013).

17. Bouazza, S., Abbouyi, S., El Kinany, S., El Rhazi, K. & Zarrouq, B. Association between Problematic Use of Smartphones and Mental Health in the Middle East and North Africa (MENA) Region: A Systematic Review. *Int J Environ Res Public Health* 20, 2891 (2023).

18. Substance Abuse and Mental Health Services Administration. National Survey on Drug Use and Health (NSDUH). . https://www.samhsa.gov/data/data-we-collect/nsduh-national-survey-drug-use-and-health.

19. Blevins, C. A., Weathers, F. W., Davis, M. T., Witte, T. K. & Domino, J. L. The Posttraumatic Stress Disorder Checklist for DSM-5 (PCL-5): Development and Initial Psychometric Evaluation. *J Trauma Stress* 28, 489–98 (2015).

20. Weathers, F. W. *et al.* *The PTSD Checklist for DSM-5 (PCL-5).* . https://www.ptsd.va.gov/professional/assessment/adult-sr/ptsd-checklist.asp (2013).

21. Forkus, S. R. *et al.* The Posttraumatic Stress Disorder (PTSD) Checklist for DSM-5: A Systematic Review of Existing Psychometric Evidence. *Clin Psychol (New York)* 30, 110–121 (2023).

22. Derogatis, L. R. *The Brief Symptom Inventory.* . (Clinical Psychometric Research, Baltimore, MD, 1975).

23. Derogatis, L. R. *BSI Brief Symptom Inventory. Administration, Scoring, and Procedures Manual* . (National Computer Systems, Minneapolis, MN, 1993).

24. Serpa, A. L. de O. *et al.* Psychometric properties of the Brief Symptom Inventory support the hypothesis of a general psychopathological factor. *Trends Psychiatry Psychother* (2022) doi:10.47626/2237-6089-2021-0207.

25. Rosen, C. S. *et al.* Sixand Ten-Item Indexes of Psychological Distress Based on the Symptom Checist-90. *Assessment* 7, 103–111 (2000).

26. Spitzer, R. L., Kroenke, K., Williams, J. B. W. & Löwe, B. A Brief Measure for Assessing Generalized Anxiety Disorder. *Arch Intern Med* 166, 1092 (2006).

27. Kroenke, K., Spitzer, R. L. & Williams, J. B. W. The PHQ-9: validity of a brief depression severity measure. *J Gen Intern Med* 16, 606–613 (2001).

28. Sheehan, D. V, Harnett-Sheehan, K. & Raj, B. A. The measurement of disability. *Int Clin Psychopharmacol* 11, 89–95 (1996).

29. Tedeschi, R. G. & Calhoun, L. G. The Posttraumatic Growth Inventory: Measuring the positive legacy of trauma. *J Trauma Stress* 9, 455–471 (1996).

30. Tedeschi, R. G., Shakespeare-Finch, J., Taku, K. & Calhoun, L. G. *Posttraumatic Growth: Theory, Research, and Applications*. (Routlidge, 2018).

31. World Health Organization. *Adverse Childhood Experiences International Questionnaire*. https://www.who.int/publications/m/item/adverse-childhood-experiences-international-questionnaire-(ace-iq) (2018).

32. Zimet, G. D., Dahlem, N. W., Zimet, S. G. & Farley, G. K. The Multidimensional Scale of Perceived Social Support. *J Pers Assess* 52, 30–41 (1988).

33. Dambi, J. M. *et al.* A systematic review of the psychometric properties of the cross-cultural translations and adaptations of the Multidimensional Perceived Social Support Scale (MSPSS). *Health Qual Life Outcomes* 16, 80 (2018).

34. Campbell-Sills, L. & Stein, M. B. Psychometric analysis and refinement of the Connor-davidson Resilience Scale (CD-RISC): Validation of a 10-item measure of resilience. *J Trauma Stress* 20, 1019–28 (2007).

35. Victor, S. E. & Klonsky, E. D. Validation of a Brief Version of the Difficulties in Emotion Regulation Scale (DERS-18) in Five Samples. *J Psychopathol Behav Assess* 38, 582–589 (2016).

36. Rammstedt, B. & John, O. P. Measuring personality in one minute or less: A 10-item short version of the Big Five Inventory in English and German. *J Res Pers* 41, 203–212 (2007).

37. Leykin, D., Lahad, M., Cohen, O., Goldberg, A. & Aharonson-Daniel, L. Conjoint Community Resiliency Assessment Measure‐28/10 Items (CCRAM28 and CCRAM10): A Self‐report Tool for Assessing Community Resilience. *Am J Community Psychol* 52, 313–323 (2013).

38. Kimhi, S. & Eshel, Y. Measuring national resilience: A new short version of the scale (NR‐13). *J Community Psychol* 47, 517–528 (2019).

39. Kimhi, S., Eshel, Y., Lahad, M. & Leykin, D. National Resilience: A New Self-Report Assessment Scale. *Community Ment Health J* 55, 721–731 (2019).

40. Carver, C. S. You want to measure coping but your protocol’s too long: consider the brief COPE. *Int J Behav Med* 4, 92–100 (1997).

41. Steinberg, L., Sharp, C., Stanford, M. S. & Tharp, A. T. New tricks for an old measure: the development of the Barratt Impulsiveness Scale-Brief (BIS-Brief). *Psychol Assess* 25, 216–26 (2013).

42. Babor, T. F., Higgins-Biddle, J. C., Saunders, J. B. & Monteiro, M. G. *The Alcohol Use Disorders Identification Test Guidelines for Use in Primary Care*. (WHO, Geneva, 2001).

43. Yudko, E., Lozhkina, O. & Fouts, A. A comprehensive review of the psychometric properties of the Drug Abuse Screening Test. *J Subst Abuse Treat* 32, 189–98 (2007).

44. Reid, R. C., Garos, S. & Carpenter, B. N. Reliability, Validity, and Psychometric Development of the Hypersexual Behavior Inventory in an Outpatient Sample of Men. *Sex Addict Compulsivity* 18, 30–51 (2011).

45. Bőthe, B. *et al.* The Psychometric Properties of the Hypersexual Behavior Inventory Using a Large-Scale Nonclinical Sample. *J Sex Res* 56, 180–190 (2019).

46. Levin, Y. *et al.* The association between type of trauma, level of exposure and addiction. *Addictive behaviors* 118, 106889 (2021).

47. Lafontaine, M.-F. *et al.* Selecting the Best Items for a Short-Form of the Experiences in Close Relationships Questionnaire. *European Journal of Psychological Assessment* 32, 140–154 (2016).

49. Robins, R. W., Hendin, H. M. & Trzesniewski, K. H. *Single-Item Self-Esteem Scale*. (2001) doi:10.1037/t16250-000.

50. Buysse, D. J., Reynolds, C. F., Monk, T. H., Berman, S. R. & Kupfer, D. J. The Pittsburgh Sleep Quality Index: a new instrument for psychiatric practice and research. *Psychiatry Res* 28, 193–213 (1989).

51. Fabbri, M. *et al.* Measuring Subjective Sleep Quality: A Review. *Int J Environ Res Public Health* 18, (2021).

52. Shochat, T., Tzischinsky, O., Oksenberg, A. & Peled, R. Validation of the Pittsburgh Sleep Quality Index Hebrew translation (PSQI-H) in a sleep clinic sample. *Isr Med Assoc J* 9, 853–6 (2007).

53. Weekers, L. C., Hutsebaut, J. & Kamphuis, J. H. The Level of Personality Functioning Scale‐Brief Form 2.0: Update of a brief instrument for assessing level of personality functioning. *Personal Ment Health* 13, 3–14 (2019).

54. Weekers, L. C., Sellbom, M., Hutsebaut, J., Simonsen, S. & Bach, B. Normative data for the LPFS-BF 2.0 derived from the Danish general population and relationship with psychosocial impairment. *Personal Ment Health* 17, 157–164 (2023).

55. Steger, M. F., Frazier, P., Oishi, S. & Kaler, M. The meaning in life questionnaire: Assessing the presence of and search for meaning in life. *J Couns Psychol* 53, 80–93 (2006).

56. Jovanović, V., Ilić, M., Šakan, D. & Brdar, I. The Meaning in Life Questionnaire: Revisiting the Evidence of Validity and Measurement Invariance Using the Exploratory Structural Equation Modeling. *Assessment* 10731911241304224 (2024) doi:10.1177/10731911241304223.

58. Brugha, T. S. & Cragg, D. The List of Threatening Experiences: the reliability and validity of a brief life events questionnaire. *Acta Psychiatr Scand* 82, 77–81 (1990).

59. Brugha, T., Bebbington, P., Tennant, C. & Hurry, J. The List of Threatening Experiences: a subset of 12 life event categories with considerable long-term contextual threat. *Psychol Med* 15, 189–94 (1985).

60. Wickwire, E. M., Burke, R. S., Brown, S. A., Parker, J. D. & May, R. K. Psychometric evaluation of the National Opinion Research Center DSM-IV Screen for Gambling Problems (NODS). *Am J Addict* 17, 392–5 (2008).

61. Bőthe, B. *et al.* The Development of the Problematic Pornography Consumption Scale (PPCS). *J Sex Res* 55, 395–406 (2018).

62. Karila, L. *et al.* Understanding Problematic Smartphone and Social Media Use Among Adults in France: Cross-Sectional Survey Study. *JMIR Ment Health* 12, e63431–e63431 (2025).

63. Greenfield, D. Phone Addiction Test. https://virtual-addiction.com/smartphone-compulsion-test/ (2025).

64. Gray, M. J., Litz, B. T., Hsu, J. L. & Lombardo, T. W. Psychometric Properties of the Life Events Checklist. *Assessment* 11, 330–341 (2004).

65. Weathers, F. W. . *et al.* The Life Events Checklist for DSM-5 (LEC-5). . *National Center for PTSD* (2013).

66. Hoyle, R. H., Stephenson, M. T., Palmgreen, P., Lorch, E. P. & Donohew, R. L. Reliability and validity of a brief measure of sensation seeking. *Pers Individ Dif* 32, 401–414 (2002).

67. Jones, D. N. & Paulhus, D. L. Introducing the Short Dark Triad (SD3). *Assessment* 21, 28–41 (2014).

68. Hunsley, J., Best, M., Lefebvre, M. & Vito, D. The Seven-Item Short Form of the Dyadic Adjustment Scale: Further Evidence for Construct Validity. *Am J Fam Ther* 29, 325–335 (2001).

69. Russell, D. W. UCLA Loneliness Scale (Version 3): Reliability, Validity, and Factor Structure. *J Pers Assess* 66, 20–40 (1996).

70. Levenson, H. Multidimensional locus of control in psychiatric patients. *J Consult Clin Psychol* 41, 397–404 (1973).

**Table S2.**

Measures for Each of Measurement in the Current Study.

|  |  | **April 2022** | **December 2023** | **March 2024** | **June 2024** |
| --- | --- | --- | --- | --- | --- |
| Sociodemographics – age, gender, area of residence, religiosity, education, ethnicity, marital status, work status, children, economic status, army service (in long study, separate section) | In-house | Also immigrant status, kupat cholim | Also physical health, sexual identity; change in kids' framework, change in work; no immigrant status or kupat cholim | Only: marital status, kids 0-18, economic status, physical health; also change in work/(kids) school since Oct 7 | Only: marital status, economic status, physical health; also change in work/school (kids) since Oct 7 |
| *Substance addictions (non-medical use)* |  |  |  |  |  |
| Alcohol | AUDIT | YES | NO | NO | NO |
| Alcohol | ASSIST 3.1 | YES | YES | YES | YES |
| Binge drinking | Adapted AUDIT Q3 | NO | NO | NO | NO |
| Tobacco | ASSIST 3.1 | YES | YES | YES | YES |
| Number of cigarettes | Adapted FTND | NO | NO | NO | NO |
| Cannabis | ASSIST 3.1 | YES | YES | YES | YES |
| Sedatives | ASSIST 3.1 | YES | YES (specified prescription) | YES (specified prescription) | YES (specified prescription) |
| Cocaine | ASSIST 3.1 | YES | NO | NO | NO |
| Amphetamines | ASSIST 3.1 | YES | NO | NO | NO |
| Hallucinogens | ASSIST 3.1 | YES | NO | NO | NO |
| Opioids (heroin, morphine, codeine, etc) | ASSIST 3.1 | YES | NO | NO | NO |
| Inhalants | ASSIST 3.1 | YES | NO | NO | NO |
| Prescription opioid painkillers | ASSIST 3.1 | YES, separate module | YES | YES | YES |
| Prescription stimulants | ASSIST 3.1 | YES, separate module | YES | YES | YES |
| Any drug (not alcohol or tobacco) | DAST-10 | YES | NO | NO | NO |
| *Behavioral Addictions* |  |  |  |  |  |
| Gambling | NODS | YES | NO | NO | NO |
| Gambling | PGSI | YES | YES | YES | YES |
| Gaming | GAS | YES | YES | YES | YES |
| Compulsive sexual behavior | HBI | YES | NO | NO | NO |
| Compulsive sexual behavior | BYSAS | YES | YES | YES | YES |
| Paying for sex | In-house | NO | NO | YES | YES |
| Pornography | PPCS | YES | NO | NO | NO |
| Pornography | PPUS | YES | YES | YES | YES |
| Internet | IAT | YES | YES | YES | YES |
| Internet – online activities | OLact | YES | NO | NO | NO |
| Social media | BSMAS | YES | YES | YES | YES |
| Smartphone | SCT | YES | NO | NO | NO |
| Cell activities | CELL | YES | NO | NO | NO |
| Smartphone | SAS-SV | NO | YES | YES | YES |
| Duration social media, news | In-house | NO | NO | YES | YES |
| *Substance and behavioral addictions* |  |  |  |  |  |
| Subjective– problematic use/disorder | In-house | NO | YES | NO | NO |
| change in use, before/after Oct 7 | In-house | NO | YES | YES | NO |
| treatment | In-house | YES | YES (different from 2022/25) | NO | NO |
| *Psychopathology* |  |  |  |  |  |
| PTSD | PCL | YES | YES | YES | YES |
| General | BSI | Long version | Short version SCL-10R | Short version SCL-10R | SCL-10R |
| Anxiety | GAD-7 | NO | YES | YES | YES |
| Depression | PHQ-9 | NO | YES | YES | YES |
| Mental health before Oct 7 | In-house | NO | YES | NO | NO |
| Functional impairment | SDS | NO | YES | YES | YES |
| Post-traumatic growth | PTGI | NO | YES | YES | NO |
| *Trauma/stress* |  |  |  |  |  |
| Adverse childhood experiences | WHO ACE-IQ | NO | YES | NO | NO |
| Oct 7 exposure | In-house | NO | YES | NO | NO |
| Ongoing stressors since Oct 7^a^ | In-house | NO | YES | YES | YES |
| Traumatic/ stressful events | LTE | YES | NO | NO | NO |
| Early childhood- family economics | ELS | YES – also family history addiction, mental health | NO | NO | NO |
| *Risk/protective factors* |  |  |  |  |  |
| Social support | MSPSS | YES | YES | YES | NO |
| Personal resilience | CD-RISC | NO | YES | YES | YES |
| Emotional regulation | DERS | YES (18 items) | YES (18 items) | YES (18 items) | YES (18 items) |
| Personality (Big 5) | BFI | NO | NO | Short version (BFI-10) | NO |
| Community resilience | CCRAM-10 | NO | YES | YES | YES |
| National resilience | NR-13 | NO | YES | NO | YES (NR-16) |
| Coping mechanisms | COPE | NO | NO | YES | NO |
| Impulsivity | BIS | NO | NO | YES | YES |
| Attachment | ECR | NO | NO | NO | YES |

Refugee status, rocket attacks, ongoing exposure to war, feelings of danger, changes in work, children at home, army/security service, financial insecurity, hate speech on social media; in 2024, added school/career insecurity, tension in personal relationships, and time spent on social media, news [some minor differences in wording across time point.

**Appendix 3: The distribution of Problem Gambling in the Sample**

**Table S3.**

*The distribution of Problem Gambling Risk Categories in 2022 By Gender (N = 899).*

|  | **Non-Problem** | **Low Risk** | **Moderate Risk** | **Problem Gamblers** |
| --- | --- | --- | --- | --- |
| Men | 366 (80.4%) | 46 (10.1%) | 27 (5.9%) | 16 (3.5%) |
| Women | 401 (90.3%) | 27 (6.1%) | 8 (1.8%) | 8 (1.8%) |
